# Supplementary material for: Evaluating spatially adaptive guidelines for the treatment of gonorrhea to reduce the incidence of gonococcal infection and increase the effective lifespan of antibiotics
Source: PLoS Comput Biol. 2022 Feb 9;18(2):e1009842. doi: 10.1371/journal.pcbi.1009842 (PMC8863219; doi:10.1371/journal.pcbi.1009842)
Supplement: S1 Text — Table A: Estimated population of men who have sex with men (MSM) and the rate of reported gonorrhea cases per 100,00 MSM population in 16 U.S. metropolitan areas. Table B: Prior distributions and posterior intervals of model parameters that are assumed to be the same across all metropolitan areas. Table C: Uncertainty range and feasible intervals of model parameters related to resistance emergence and spread (assumed to be the same across all metropolitan areas). Table D: Prior distributions and posterior intervals for initial gonorrhea prevalence and initial proportion of gonorrhea that are symptomatic in each metropolitan area. Table E: Uncertainty range and feasible intervals for the initial prevalence of resistance to Drug A or Drug B in each metropolitan area. Table F: Uncertainty range and feasible intervals for the annual importation rate of cases resistant to Drug A or Drug B in each metropolitan area Table G: Prior distributions selected in the primary and sensitivity analyses Table H: Uncertainty ranges selected in the primary and sensitivity analyses Table I: Correlation between select model input parameters and the effective lifespan of drugs A and B under the ‘Base’ strategy. Table J: Correlation between select model input parameters and the change in the effective lifespan of drugs A and B under the ‘Spatial’ strategy with respect to the ‘Base’ strategy. Fig A: Expanded model of gonorrhea transmission among the MSM population in 16 metropolitan areas in the United States. Fig B: Behavior of function γ(t) (defined in Eq (2)) over time. Fig C: The rate of gonorrhea cases per 100,000 MSM population in 100 simulated runs compared with the estimated rate of gonorrhea cases among the MSM population in 2017 (as shown by green dot). Fig D: The prevalence of gonorrhea among the MSM population of the metropolitan areas included in our model. Fig E: Number of gonorrhea cases treated successfully with Drugs A or B, and Drug M during the simulation year. Fig F: The [file pcbi.1009842.s001.pdf]

S1 Text: Additional model details and results of sensitivity analyses

S1 Additional model details

S1.1 Model population

We developed a stochastic compartmental model to simulate the transmission of gonorrhea among men who have sex with me (MSM) of age 14 or older in 16 U.S. metropolitan areas (Fig. 1 and Table A). We assumed that an individual stays in the model for an average of 35 years (representing the period when an individual could be sexually active). The estimated population size of MSM and the estimated rate of gonorrhea cases in these areas are provided in Table A.

Table A: Estimated population of men who have sex with men (MSM) and the rate of reported gonorrhea cases per 100,00 MSM population in 16 U.S. metropolitan areas.

Metropolitan Area	Estimated MSM Population (2013) [1]	Estimated Rate of Reported Gonorrhea Cases per 100,000 MSM Population (2017)‡
Atlanta-Sandy Springs-Roswell	102,642	6,516
Boston-Cambridge-Newton	92,527	2,790
Chicago-Naperville-Elgin	175,118	5,949
Dallas-Fort Worth-Arlington	133,944	5,416
Houston-The Woodlands-Sugar Land	103,722	4,858
Los Angeles-Long Beach-Anaheim	313,711	6,797
Miami-Fort Lauderdale-West Palm Beach	141,088	4,449
Minneapolis-St. Paul-Bloomington	71,099	4,516
New York-Newark-Jersey City	397,399	4,845¶
Philadelphia-Camden-Wilmington	100,293	6,077¶
Phoenix-Mesa-Scottsdale	92,825	6,172
Riverside-San Bernardino-Ontario	94,863	4,937
San Diego-Carlsbad	80,968	5,489
San Francisco-Oakland-Hayward	145,972	7,931¶
Seattle-Tacoma-Bellevue	82,002	5,352
Washington-Arlington-Alexandria	122,895	4,815
All	2,251,068	5,285

‡ To estimate the rate of reported gonorrhea cases per 100,000 MSM population, we multiply the rates of reported gonorrhea cases in these metropolitan areas by the ratio 5241.8/171.9 (5241.8 is the estimated rate of reported gonorrhea cases per 100,000 MSM in 2017 and 171.9 is the rate of reported gonorrhea cases per 100,000 U.S population in 2017 [2].
¶ The rate of reported gonorrhea cases for these cities were obtained from [3].

S1.2 Simulation approach

To construct the model, we introduce the following notation (adapted from [4]):

- $k \in \{1,2,\dots,16\}$: index of metropolitan areas.
- $i \in \{0, A, B, AB\}$: resistance profile an infection ($i = 0$, drug-susceptible; $i = A$, resistance to Drug A; $i = B$, resistance to Drug B; and $i = AB$, resistance to both Drug A and Drug B);
- $s \in \{0,1\}$: symptom status ($i = 0$, asymptomatic, and $i = 1$, symptomatic);
- t: epidemic time;
- $N_k(t)$: population size of metropolitan area k at time t;
- $S_k(t)$: number of susceptibles in metropolitan area k at time t;
- $I_{(k,i,s)}(t)$: number of infected cases in metropolitan area k with resistance profile i and symptom status s at time t;
- $W_{(k,i,s)}(t)$: number of diagnosed cases with the resistance profile i, waiting to receive the first-line therapy in the metropolitan area k at time t;
- $W'_{(k,i,s)}(t)$: number of diagnosed cases with the resistance profile i, waiting to receive the second-line therapy in the metropolitan area k at time t.

The state of the gonorrhea epidemic at any given time t can be identified by a discrete-time Markov chain $\{(S_k(t), I_{(k,i,s)}(t), W_{(k,i,s)}(t), W'_{(k,i,s)}(t), \mathrm{k} \in \{1,2,\dots,16\}, i \in \{0, A, B, AB\}, s \in \{0,1\}: t = 0, \Delta t, 2\Delta t, 3\Delta t, \dots\}$, where Δt is the time-step of the simulation (e.g. $\Delta t = 1$ day). To generate epidemic trajectories for this model, we use Monte Carlo simulation to sample from this Markov chain using the following approach. Consider a particular compartment Z in which members depart due to J events each of which is occurring at the rate $\mu_j, j \in \{1,2,\dots,J\}$. For example, members of Susceptible compartment may leave due to 1) infection with the susceptible strain, 2) infection with Drug-A resistant strain, 3) infection with Drug-B resistant strain, or 4) infection with a strain resistant to both drugs (i.e. $J = 4$) (see Fig. 1). If the number of individuals in compartment Z at time t is $Z(t)$, then the number of individuals that leave this compartment due to events $j \in \{1,2,\dots,J\}$ follows a multinomial distribution with total counts of $Z(t)$ and probabilities $(p_0, p_1, p_2, \dots p_J)$, where $p_0 = 1 - e^{\sum_{j=1}^{J} \mu_j \Delta t}$ is the probability of not leaving the compartment Z during $[t, t + \Delta t]$, and $p_j = \frac{\mu_j}{\sum_{j=1}^{J} \mu_j \Delta t} e^{\sum_{j=1}^{J} \mu_j \Delta t}$ is the probability of leaving the compartment Z during $[t, t + \Delta t]$ due to the event $j \in \{1,2,\dots,J\}$.

To identify the new epidemic state at the next time step, we first sample from the multinomial distributions associated to each compartment and then use these realizations to calculate the new epidemic state given the current epidemic state. The events that drive the epidemic are represented by black arrows in Fig. 1. For example, the number of susceptibles in metropolitan area k at time $t + \Delta t$ can be calculated as:

$$S_k(t + \Delta t) = S_k(t)$$

$-$ new infections susceptible to Drugs A and B in city k

$-$ new infections resistant to Drug A in city k

$-$ new infections resistant to Drug B in city k

$-$ new infections resistant to Drug A and Drug B in city k

$-$ members leaving city k

$+$ new population members to city k

$+$ members recovering from infection in city k.

Number of members in other model compartments (i.e. $I_{(k,i,s)}(t)$, $W_{(k,i,s)}(t)$, and $W'_{(k,i,s)}(t)$) are updated according to the following equations. For number of population members infected with symptom and resistance profile (i, s) in city k:

$I_{(k,i,s)}(t + \Delta t) = I_{(k,i,s)}(t)$

$+$ new infections with symptom and suceptibility profile (i, s) in city k

$-$ number naturally recover from infection in city k

$-$ number seeking treatment in city k

$-$ number detected through screening in city k.

For number of population members waiting to receive first-line treatment with symptom and resistance profile (i, s) in city k:

$W_{(k,i,s)}(t + \Delta t) = W_{(k,i,s)}(t)$

$+$ members with symptom and suceptibility profile (i, s) seeking treatment in city k

$+$ members with symptom and suceptibility profile (i, s) detected through screening in city k

$-$ number receiving first-line treatment in city k

For number of population members waiting to receive second-line treatment with symptom and resistance profile (i, s) in city k:

$W'_{(k,i,s)}(t + \Delta t) = W'_{(k,i,s)}(t)$

$+$ number with symptom and suceptibility profile (i, s)

failing first-line therapy and seeking retreatment in city k

$-$ number receiving second-line treatment in city k

We note that in updating $I_{(k,i,s)}(t)$ based on the above equation, "new infections with symptom and resistance profile (i, s)" include 1) susceptibles who become infected according to the force-of-infection model described below, and 2) those who experience the emergence of resistance during the first-line therapy (Fig A).

To update $W_{(k,i,s)}(t)$ and $W'_{(k,i,s)}(t)$, we made the following assumptions:

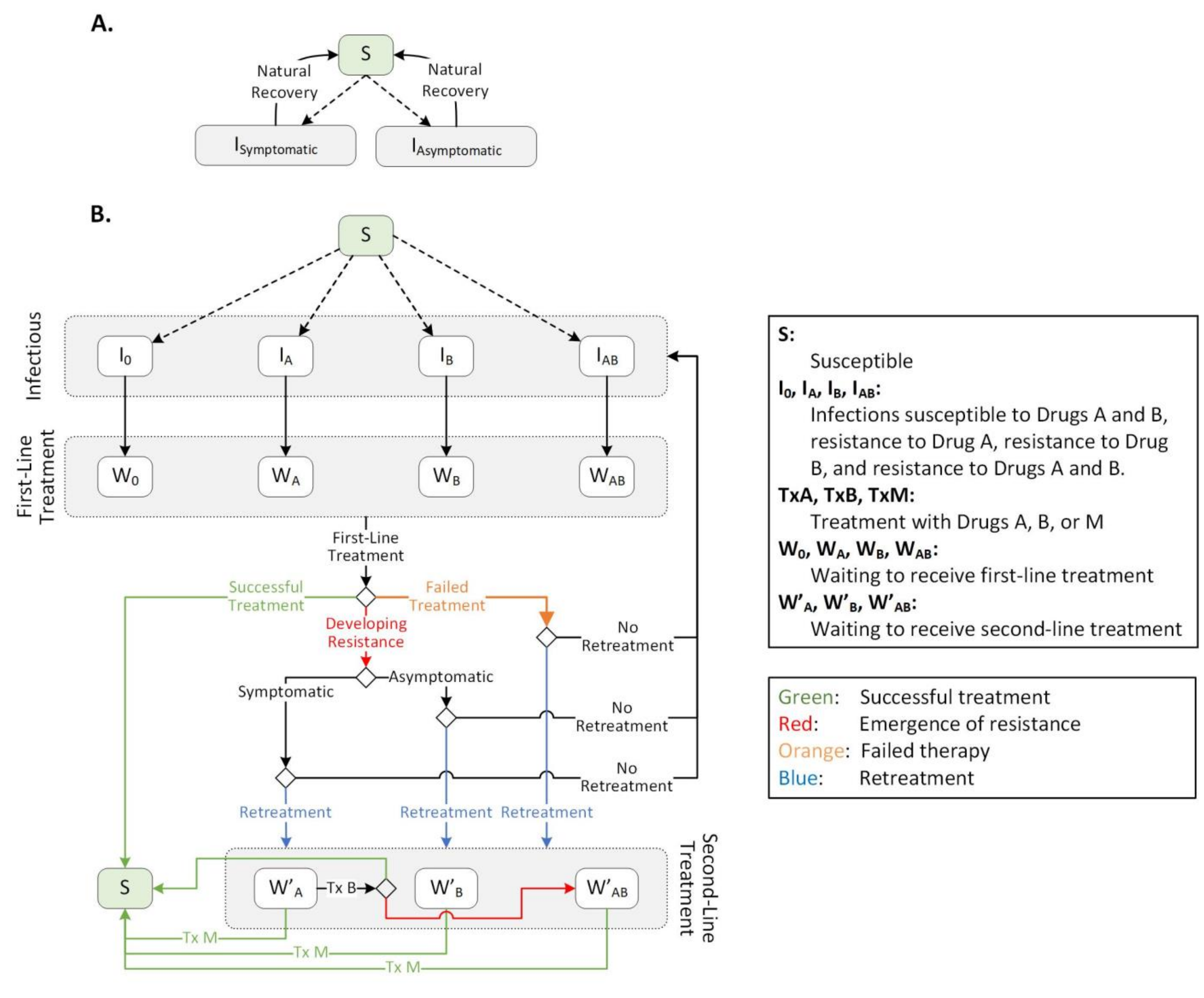

Fig A: Expanded transmission dynamic model of gonorrhea among the MSM population in 16 metropolitan areas in the United States.

- Asymptomatic cases do not seek treatment and remain infectious until they recover spontaneously or get detected through active screening.
- Symptomatic cases who seek treatment and asymptomatic cases who are detected through screening will receive treatment with either Drug A, B, or M, depending on the current recommendation for first-line therapy.
- Treatment always fails if the infecting strain is resistant to the prescribed antibiotic.
- Treatment with an antibiotic to which the infecting strain is susceptible may lead to one of two possible outcomes: 1) the treatment succeeds and the individual returns to the susceptible state, or 2) the treatment leads to resistance and fails.

- A fraction of symptomatic individuals failing the first-line treatment (due to receiving ineffective treatment or developing resistance) will seek retreatment with some delay. These individuals will receive a second-line antibiotic which is Drug B in case of resistance to Drug A, and is Drug M in case of resistance to Drug B.
- We assume that treatment with Drug M is always successful but treatment with Drug B could lead to the selection of resistance with a small probability, upon which the individual will be treat with Drug M
- Those symptomatic individuals who fails the first-line treatment but do not seek retreatment will remain in their current state where they may naturally recover or seek treatment in future time steps.

The model is coded in C# (release 8) and the analyses were done in Python (version 3.8).

S1.3 Calculating the rate of infection

We calculate the weekly rate of infection with resistance profile $i \in \{0, A, B, AB\}$ in metropolitan area k at time t as:

$$\mathcal{F}_{k,i}(t) = \beta_{k,i}(t) \sum_{s \in \{0,1\}} \frac{I_{(k,i,s)}(t) + W_{(k,i,s)}(t) + W'_{(k,i,s)}(t)}{N_k(t)}, \quad (1)$$

where $\beta_{k,i}(t)$ is the transmission parameter for resistance profile $i \in \{0, A, B, AB\}$ in metropolitan area k. We assumed $\beta_{k,i}(t) = \gamma_i(t)\beta$ for $k \in \{1,2, \dots, 16\}$ and $i \in \{A, B, AB\}$, where $\beta \geq 0$ is the transmission parameter that is determined through calibration (as described in §S3) and $0 \leq \gamma_i(t) \leq 1$ represents the fitness cost associated with the resistance profile $i \in \{A, B, AB\}$. To allow fitness cost to decrease over time, we let the relative infectiousness of the resistance profile $i \in \{A, B, AB\}$ increase over time according to:

$$\gamma_i(t) = b_{i,min} + \frac{1 - b_{i,min}}{1 + e^{-b_i(t - t_{i,0})}}. \quad (2)$$

Here, $b_{i,min} \geq 0$, $b_i \geq 0$, and $t_{i,0} \geq 0$. Fig B displays how $\gamma(t)$ changes over time and how the parameters of this function (i.e. $b_{i,min}$, b_i, and $t_{i,0}$) impact this behavior. These parameters are determined through the calibration procedure described below.

S1.4 Importation of drug-resistant cases

We also allowed for the importation of resistant cases to each metropolitan area that occurs continuously over time according to a Poisson process with rate parameters provided in Table F.

S2 Sampling error in estimating the resistance prevalence

The decision about which antibiotic to include in the first-line treatment recommendation is based on estimates of resistance prevalence obtained from surveillance systems, such as GISP [5], by evaluating a limited number of gonorrhoeae isolates for drug susceptibility. Hence, the estimates of resistance prevalence are affected by sampling error. To account for this sampling error when evaluating the policies of Table 1 using our simulation model, we use the following approach. Let y_t be the proportion of gonorrhea cases in the simulation year t that are resistant. Since not all cases are tested for drug-susceptibility, we assumed that p_t can be observed with some noise:

$$\hat{y}_t = y_t + \epsilon_t.$$

Here we assume that ϵ_t follow a normal distribution with mean 0 and standard deviation $\sqrt{y_t(1-y_t)/N}$, where N is the number of gonorrhea cases tested for drug-susceptibility. A higher value of N decreases the variance of

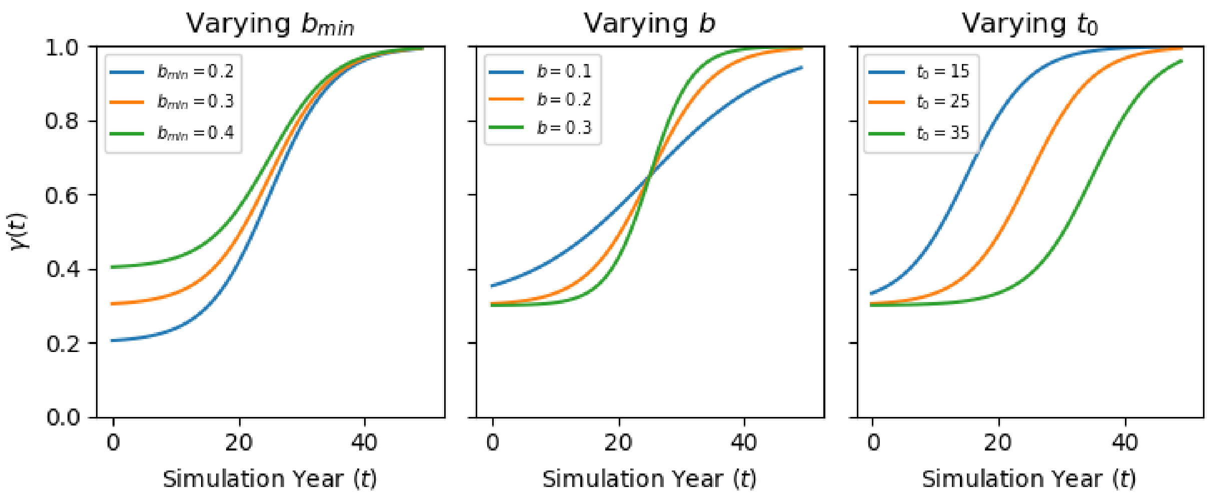

Fig B: Behavior of function $\gamma(t)$ (defined in Eq. (2)) over time. In these figures, the non-varying parameters are set at the default values $(b_{min}, b, t_0) = (0.3, 0.2, 25)$.

the error ϵ_t leading to more accurate estimates for the prevalence of resistance. Fig. 2D-F displays the estimated proportion of cases resistant to Drugs A, B or both when $N = 5{,}000$ of annual gonorrhea cases are tested for drug resistance during each simulation. This assumption is informed by how many *N. gonorrhoeae* isolates are collected and tested through GISP in 2014 (5,093 isolates) [5].

S3 Model calibration

The model is calibrated against estimates of gonorrhea prevalence (4.5% [3.6%, 5.4%] among MSM) [6], the annual gonorrhea rate in 2017 (5,241.8 cases per 100,000 MSM) [2] and in each metropolitan areas (as reported in Table A), and the proportion of gonorrhea cases with symptoms (67.9% [64.4-71.4%]) [7]. Our calibration procedures relies on the use of common random numbers to simulate epidemic trajectories [8]. This variance reduction technique is often used to improve the accuracy of the comparison between two or more alternative configurations by using the same streams of uniform random variates in simulating these alternatives. In this method, to obtain one simulated epidemic trajectory, we first specify the seed of the simulation's random number generator (RNG) object. The simulator will then use the RNG object to generate a unique stream of random numbers which will be used to both draw a sample for epidemic parameters and to generate one simulated trajectory. This approach will enable us to regenerate any desired trajectory by knowing the corresponding RNG seeds. For a given simulated trajectory, we approximate the likelihood of observations using a pseudolikelihood function that consists of three components as described below.

S3.1 Component 1: Likelihood of gonorrhea prevalence

To estimate the prevalence of gonorrhea among the U.S. MSM population, we used the data reported by Jones et al. [6] where among $\hat{S} = 2{,}075$ participants, $\hat{s} = 93$ (or 4.5%) were tested positive for rectal gonorrhea. To calculate the likelihood of observing this outcome in year t if a given simulated trajectory represents the reality, we assumed that $\hat{s}$ follows a binomial distribution with $\hat{S}$ trials and success probability τ_t, where τ_t is the prevalence of gonorrhea in year t of the simulation:

$$L_1 = \sum_{t=1}^{10} \binom{\hat{S}}{\hat{s}} \tau_t^{\hat{s}} (1 - \tau_t)^{\hat{S}-\hat{s}}.$$

We used the same approach to calculate the likelihood associated with the estimated prevalence of gonorrhea among the MSM population of Houston ($\hat{S} = 468, \hat{s} = 31$), Miami ($\hat{S} = 345, \hat{s} = 19$), New York City ($\hat{S} = 425, \hat{s} = 18$), San Francisco ($\hat{S} = 418, \hat{s} = 7$), and Washington, DC ($\hat{S} = 419, \hat{s} = 16$), as provided by [6]. We use $L_{1,k}$ for $k \in \{1,2,3,4,5\}$ to denote the likelihoods associated with the prevalence of gonorrhea among the MSM population of these 5 cities.

S3.2 Component 2: Likelihood of annual rate of reported gonorrhea cases

The estimated rates of gonorrhea cases per 100,000 MSM population of the 16 metropolitan areas included in our model are reported in Table A. We assume that the estimate of 5,548 cases of gonorrhea per 100,000 MSM (as reported in Table A) is calculated as $\hat{h}/\hat{H} \times 100{,}000$, where $\hat{h}$ is the number of gonorrhea cases observed in a sample MSM population of size $\hat{H}$. To calculate the likelihood of observing this outcome in year t if a given simulated trajectory represents the reality, we assumed that $\hat{h}$ follows a binomial distribution with $\hat{H}$ trials and success probability ρ_t, where ρ_t is the proportion of the simulated population year t that got diagnosed with gonorrhea:

$$L_2 = \sum_{t=1}^{10} \binom{\hat{H}}{h} \rho_t^{\hat{h}} (1-\rho_t)^{\hat{H}-\hat{h}}.$$

No confidence intervals were reported for this estimate. We therefore assumed that the estimated 5,548 cases of gonorrhea per 100,000 MSM was with 20% error which is equivalent to having a reported confidence interval of [4,438 – 6,657]. We approximate $\hat{H}$ in the above equation by noting that the half-length of the confidence interval for the estimated annual rate of reported gonorrhea cases is:

$$HL = 100{,}000 \times z_{\alpha/2} \sqrt{\frac{\mu(1-\mu)}{\hat{H}}},$$

where $\mu = \hat{h}/\hat{H}$ and $z_{\alpha/2}$ is the upper $\alpha/2$ critical point for the standard normal distribution. By using $HL = \frac{6657-4438}{2}$ =1109, $\alpha = 0.05$, and $\hat{h}/\hat{H}$=0.05548 in the above equation, we estimate $\hat{H}$ at 1,635.

We used the same approach to calculate the likelihood associated with the estimated rates of gonorrhea cases per 100,000 MSM population for each metropolitan area (reported in Table A). In these calculations, we assumed that the estimate of gonorrhea rates for each city is with 40% error (as opposed to the 20% error we assumed for the overall estimate of gonorrhea rate). This is to account for the fact that city-level estimates are often obtained with a smaller sample compared with the national-level estimates. The calculated 95% confidence intervals are displayed in Fig C. We use $L_{2,k}$ for $k \in \{1,2,\ldots,16\}$ to denote the likelihoods associated with the estimated rates of gonorrhea cases per 100,000 MSM population for each metropolitan area.

S3.3 Component 3: Likelihood of proportion of gonorrhea cases that are symptomatic

The estimated for the proportion of gonorrhea cases with symptoms (67.9% [64.4-71.4%]) [7] is obtained from a study where $\hat{r} = 466$ of $\hat{R} = 686$ gonorrhea cases presented symptoms. To calculate the likelihood of observing this outcome in year t if a given simulated trajectory represents the reality, we assumed that $\hat{r}$ follows a binomial distribution with $\hat{R}$ trials and success probability β_t, where β_t is the proportion of gonorrhea cases in year t of the simulation that are symptomatic:

$$L_3 = \sum_{t=1}^{10} \binom{\hat{R}}{\hat{r}} \varphi_t^{\hat{r}} (1-\varphi_t)^{\hat{R}-\hat{r}}.$$

S3.4 Total pseudolikelihood

We calculate the natural logarithm of the likelihood of observations given a simulated trajectory as:

$$\ln \mathcal{L} = \ln L_1 + \sum_{k=1}^{5} \ln L_{2,k} + \ln L_2 + \sum_{k=1}^{16} \ln L_{2,k} + \ln L_3.$$

To improve the efficiency of the calibration procedure, we terminate the simulation of a trajectory once any of the following conditions is met:

1. Gonorrhea prevalence falls out of the range [1%, 15%].
2. Annual rate of reported gonorrhea cases falls out of the range [1,000, 15,000],
3. Annual percentage of gonococcal infections that are symptomatic less than 50%.

Also, to make sure that resistance to Drugs A and B emerges during the simulation horizon (50 years), we eliminate trajectories where the prevalence of resistance to Drug A or Drug B never reached 5%.

Finally, we note that the prior distributions for a number of model parameters listed in Table B are different from the prior distributions we assumed for the model we presented in [4]. This is because the model described in our PLOS Medicine study was calibrated using the gonorrhea prevalence of 2.0% [1.2%, 2.8%] among the MSM population but the model presented here is calibrated against the gonorrhea prevalence of 4.5% [3.6%, 5.4%] among the MSM population of five major U.S. cities [6]. Also, in this study, we are using the estimated rate of gonorrhea cases among the MSM population of the 16 metropolitan areas included in our model instead of the estimates rate of gonorrhea cases among all U.S. MSM population which we used in PLOS Medicine.

S3.5 Projections and parameter estimation

To build a set of trajectories to evaluate the performance of strategies in Table 1, we first simulated enough trajectories to obtain 5,000 trajectories for which the above pseudo-likelihood function can be calculated (235,182 simulated trajectories were discarded due to violating the feasibility conditions described in above §S3.4). Each of these simulation trajectories uses parameter values that are randomly drawn from the probability distribution of epidemic parameters listed in Table B- Table F. The prior distributions are informed by estimates extracted from existing scientific literature when such estimates are available; when not available, we assumed biologically-feasible distributions. As Drug A and Drug B are hypothetical future antibiotic treatments for gonorrhea, we did not specify prior distributions for the parameters that relate to the emergence and spread of resistance to these drugs. Instead, we derived relevant ranges for these parameters (Table C and Table E-Table F) by retaining only those simulated trajectories where the prevalence of resistance to Drug A and Drug B reaches at least 5% during simulations.

Let $\ln \mathcal{L}_i$ be the total pseudolikelihood for the simulation trajectory $i \in \{1,2,..N_0\}$. We calculated the likelihood weight of this trajectory as:

$$w_i = \frac{e^{\ln \mathcal{L}_i - L_{max}}}{\sum_{j=1}^{N_0} e^{\ln \mathcal{L}_j - L_{max}}},$$

where $L_{max} = \max\{\ln \mathcal{L}_1, \ln \mathcal{L}_2, \ldots, \ln \mathcal{L}_{N_0}\}$. We note that since $\ln \mathcal{L}_i$ could be a negative number, $e^{\ln \mathcal{L}_i}$ might be calculated as 0 in computer even though mathematically, $e^{\ln \mathcal{L}_i} > 0$ for any $\ln \mathcal{L}_i > -\infty$. A common approach to mitigate this issue is to subtract L_{max} from all likelihoods $\ln \mathcal{L}_i$. This doesn't change the estimated w_i's but it makes the estimates more stable numerically.

After calculating w_i for each simulated trajectory, we draw 500 trajectories, with replacement and based on likelihood weights w_i. We used the parameter values associated with these 500 trajectories (out of the accepted 5,000 trajectories) to calculate the mean and 95% posterior intervals of model parameters (Table B and Table D). Fig G displays the posterior distribution and the correlation between select key model parameters listed in Table B.

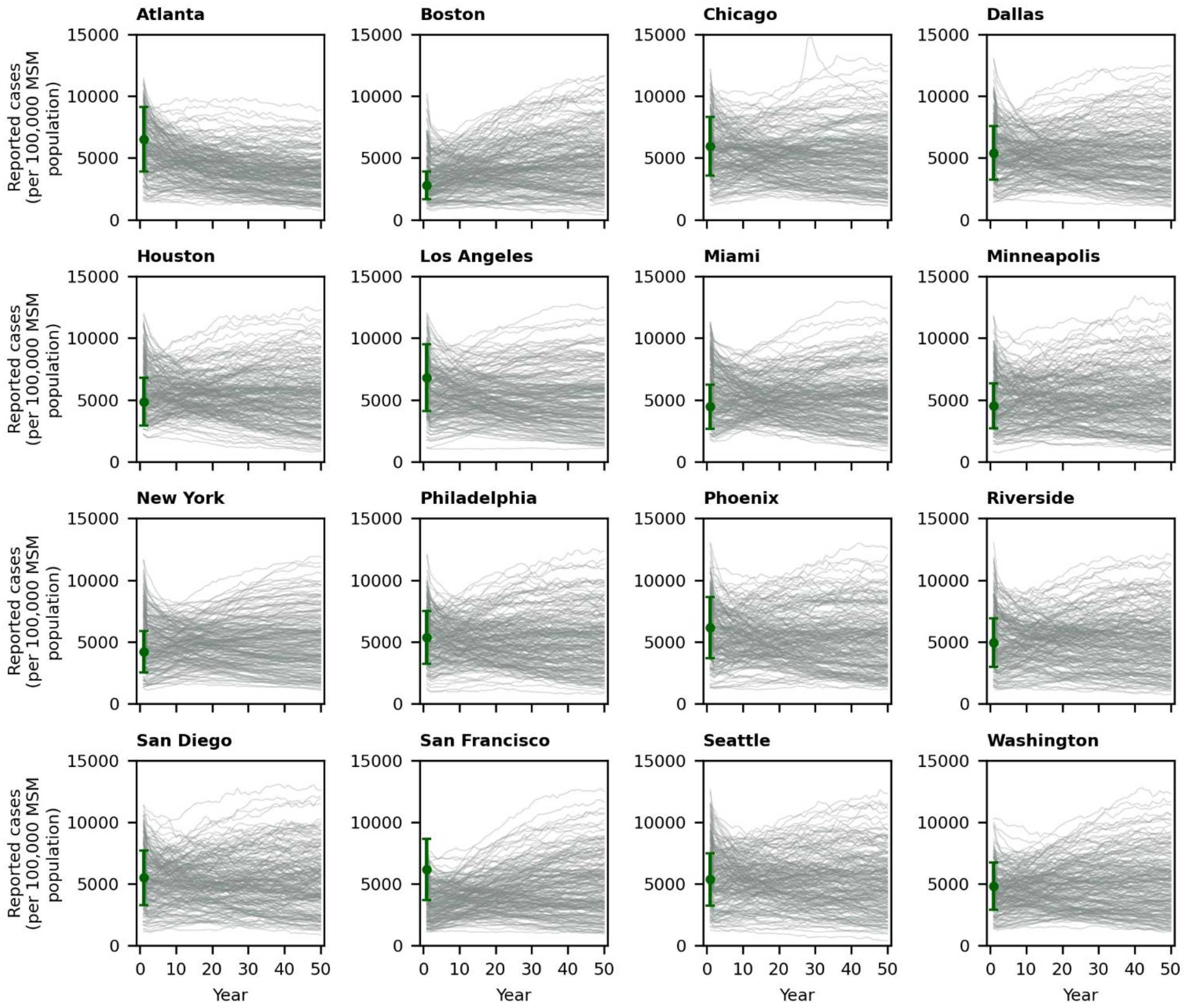

Fig C: The rate of gonorrhea cases per 100,000 MSM population in 200 simulated runs compared with the estimated rate of gonorrhea cases among the MSM population in 2017 (shown by green dot, Table A).

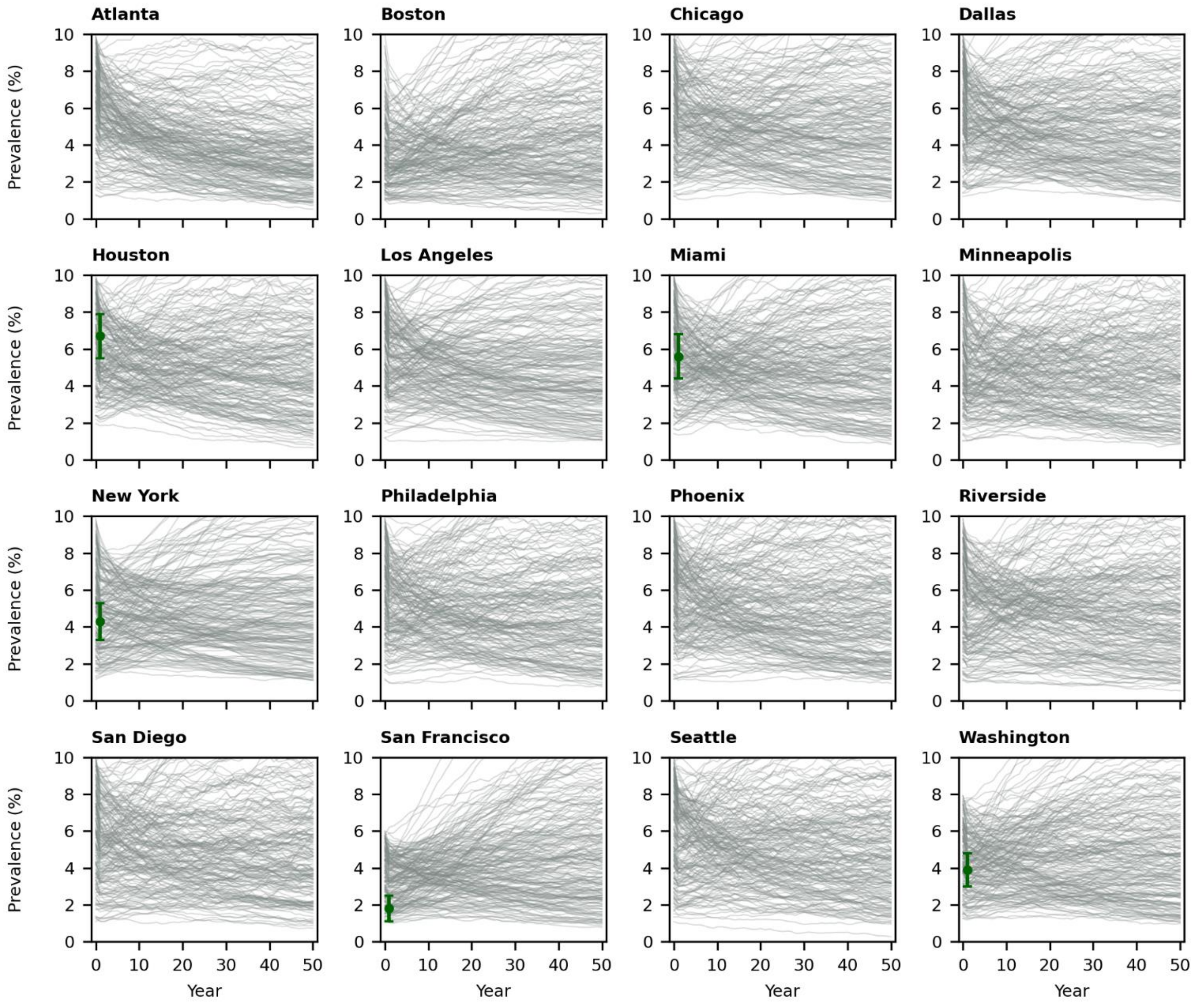

Fig D: The prevalence of gonorrhea among the MSM population of the metropolitan areas included in our model. Green dots represent the estimated prevalence of gonorrhea in 2017 for select cities reported by [6].

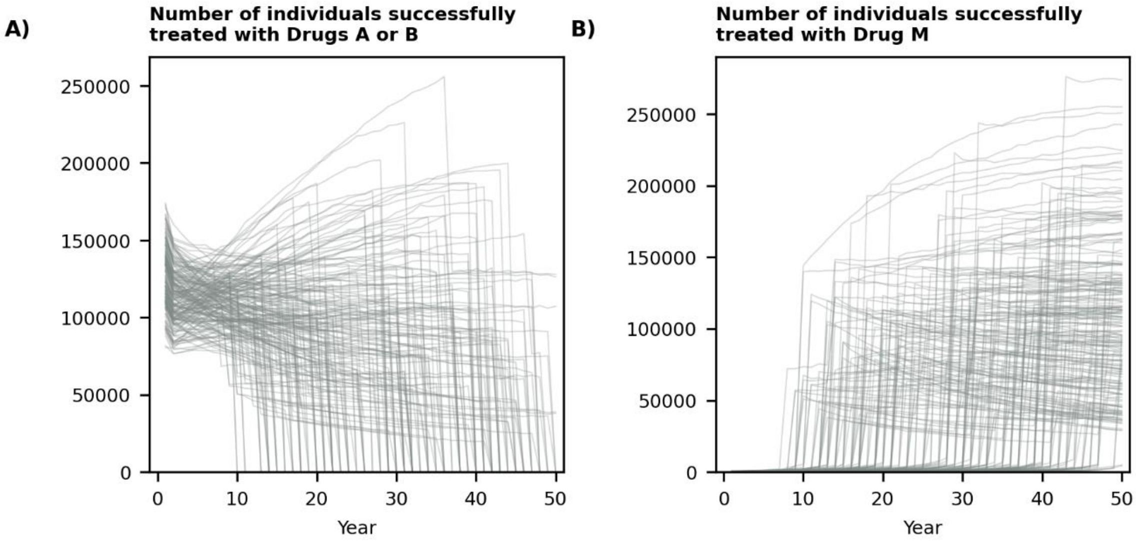

Fig E: Number of gonorrhea cases treated successfully with Drugs A or B, and Drug M during the simulation year.

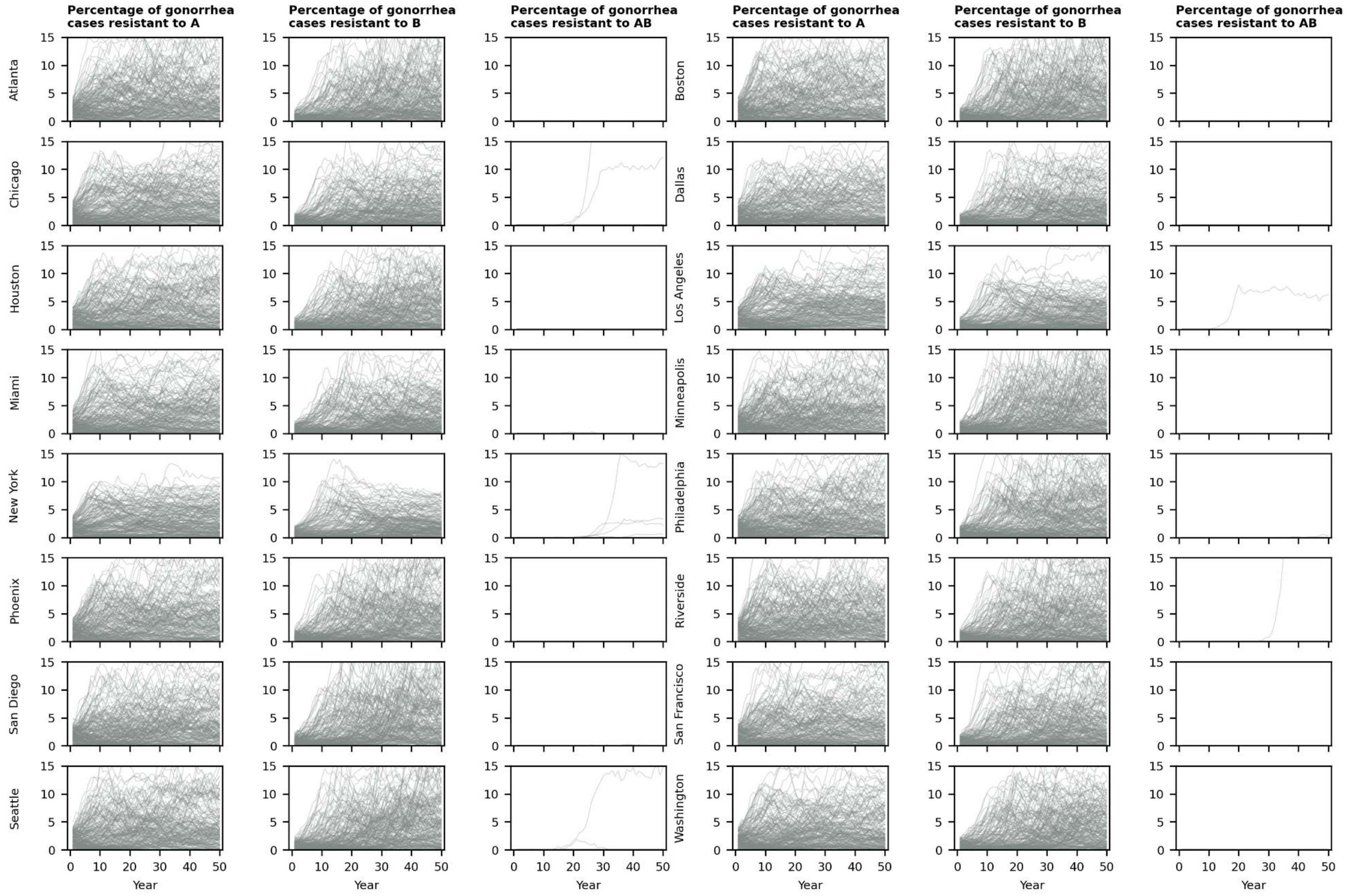

Fig F: The estimated proportion of cases resistant to Drugs A, B or both when 200 annual gonorrhea cases are tested for drug resistance in each region

Table B: Prior distributions and posterior intervals of model parameters that are assumed to be the same across all metropolitan areas.

Parameter	Prior Distribution (All Uniform)	Mean and 95% Posterior Interval	Sources to Inform Prior Distribution
Transmission parameter (β) (1/year)	[0.5, 3]	1.55 (1.06, 2.47)	Assumption
Duration of infection (without treatment) (months)	[1, 60]	37.6 (8.5, 58.7)	Assumption based on [9]
Time until screened for infection (years)	[0.5, 5.0]	3.0 (1.7, 4.7)	Assumption based on [9,10]
Time until seeking treatment for a symptomatic infection (days)	[1, 14]	7.4 (1.4, 13.5)	Assumption based on [7,9,11]
Time until retreatment (days)	[1, 14]	8.1 (1.4, 13.5)	Assumption based on [7,9]
Probability that an infection will be symptomatic	[10%, 90%]	49.2% (28.9%, 62.8%)	Assumption based on [9,10,12]
Probability of retreatment after treatment failure with symptomatic infection	[80%, 100%]	89.5% (81.3%, 98.7%)	Assumption based on [4,10]

Table C: Uncertainty range and feasible intervals of model parameters related to resistance emergence and spread (assumed to be the same across all metropolitan areas).

Parameter	Uncertainty Range (All Uniform)	Mean and 95% Feasible Interval#	Sources to Inform Uncertainty Range
Probability of developing resistance while receiving Drug A	$10^{[-6, -4]}$	$10^{-4.99}$; $10^{(-5.93, -4.07)}$	Assumption based on [4]
Probability of developing resistance while receiving Drug B	$10^{[-6, -4]}$	$10^{-4.96}$; $10^{(-5.93, -4.04)}$	Assumption based on [4]
Relative transmissibility of the strain resistant to Drug A ($\gamma_A(t)$)			Assumption
$b_{A,min}$	[0, 1]	0.66 (0.16, 0.99)	
b_A	[0, 0.2]	0.109 (0.007, 0.193)	
$t_{A,0}$	[0, 30]	12.9 (0.9, 28.2)	
Relative transmissibility of the strain resistant to Drug B or both drugs ($\gamma_B(t)$ and $\gamma_{AB}(t)$)			Assumption
$b_{B,min}$ and $b_{AB,min}$	[0, 1]	0.52 (0.05, 0.98)	
b_B and b_{AB}	[0, 0.2]	0.111 (0.010, 0.198)	
$t_{B,0}$ and $t_{AB,0}$	[0, 40]	17.8 (1.0, 38.3)"	

Mean and 95% percentile of parameter values that lead to simulated trajectories where the prevalence of resistance reaches at least 5% during the simulation.

Table D: Prior distributions and posterior intervals for initial gonorrhea prevalence and initial proportion of gonorrhea that are symptomatic in each metropolitan area

Parameter	Prior Distribution (All Uniform)	95% Posterior Interval	Sources to Inform Prior Distribution
Initial gonorrhea prevalence among the MSM population			Assumption based on [6]
Atlanta	[1%, 10%]	6.3% (1.8%, 9.7%)	
Boston	[1%, 10%]	3.8% (1.1%, 8.1%)	
Chicago	[1%, 10%]	5.9% (1.7%, 9.7%)	
Dallas	[1%, 10%]	5.9% (1.9%, 9.7%)	
Houston	[1%, 10%]	6.2% (2.4%, 9.7%)	
Los Angeles	[1%, 10%]	6.1% (2.2%, 9.8%)	
Miami	[1%, 10%]	5.9% (1.9%, 9.8%)	
Minneapolis	[1%, 10%]	5.4% (1.6%, 9.8%)	
New York	[1%, 10%]	5.3% (1.6%, 9.5%)	
Philadelphia	[1%, 10%]	5.8% (1.9%, 9.8%)	
Phoenix	[1%, 10%]	6.1% (1.4%, 9.9%)	
Riverside	[1%, 10%]	5.5% (1.5%, 9.4%)	
San Diego	[1%, 10%]	5.8% (1.4%, 9.8%)	
San Francisco	[1%, 10%]	3.6% (1.2%, 5.8%)	
Seattle	[1%, 10%]	6.0% (1.7%, 9.7%)	
Washington	[1%, 10%]	4.7% (1.5%, 7.6%)	
Initial proportion of gonorrhea that are symptomatic			Assumption based on [4]
Atlanta	[0%, 25%]	12.6% (1.0%, 24.2%)	
Boston	[0%, 25%]	13.3% (1.4%, 24.6%)	
Chicago	[0%, 25%]	12.7% (1.2%, 24.1%)	
Dallas	[0%, 25%]	13.5% (0.9%, 24.4%)	
Houston	[0%, 25%]	11.8% (0.4%, 24.2%)	
Los Angeles	[0%, 25%]	12.9% (2.2%, 24.2%)	
Miami	[0%, 25%]	13.2% (1.0%, 24.4%)	
Minneapolis	[0%, 25%]	12.6% (0.7%, 24.5%)	
New York	[0%, 25%]	12.6% (0.8%, 24.2%)	
Philadelphia	[0%, 25%]	12.4% (0.4%, 24.3%)	
Phoenix	[0%, 25%]	12.2% (0.5%, 24.6%)	
Riverside	[0%, 25%]	12.6% (0.6%, 24.0%)	
San Diego	[0%, 25%]	11.9% (0.6%, 24.4%)	
San Francisco	[0%, 25%]	12.5% (0.5%, 24.6%)	
Seattle	[0%, 25%]	12.1% (1.0%, 23.8%)	
Washington	[0%, 25%]	11.9% (0.5%, 24.1%)	

Table E: Uncertainty range and feasible intervals for the initial prevalence of resistance to Drug A or Drug B in each metropolitan area

Parameter	Uncertainty Range (All Uniform)	Mean and 95% Feasible Interval#	Sources to Inform Uncertainty Ranges
Initial prevalence of resistance to Drug A			Assumptions based on [5,13]‡
Atlanta	[0%, 4%]	2.1% (0.2%, 3.9%)	
Boston	[0%, 4%]	2.1% (0.1%, 3.9%)	
Chicago	[0%, 4%]	2.1% (0.1%, 3.9%)	
Dallas	[0%, 4%]	1.9% (0.1%, 4.0%)	
Houston	[0%, 4%]	1.9% (0.1%, 3.9%)	
Los Angeles	[0%, 4%]	2.1% (0.1%, 3.9%)	
Miami	[0%, 4%]	1.9% (0.1%, 3.9%)	
Minneapolis	[0%, 4%]	2.1% (0.2%, 3.9%)	
New York	[0%, 4%]	1.9% (0.1%, 3.8%)	
Philadelphia	[0%, 4%]	1.9% (0.2%, 3.7%)	
Phoenix	[0%, 4%]	2.1% (0.1%, 3.9%)	
Riverside	[0%, 4%]	2.2% (0.2%, 3.9%)	
San Diego	[0%, 4%]	2.0% (0.2%, 3.8%)	
San Francisco	[0%, 4%]	2.0% (0.1%, 3.9%)	
Seattle	[0%, 4%]	2.1% (0.1%, 3.9%)	
Washington	[0%, 4%]	2.0% (0.1%, 3.9%)	
Initial prevalence of resistance to Drug B			Assumption based on [5,13]‡
Atlanta	[0%, 2%]	1.0% (0.1%, 2.0%)	
Boston	[0%, 2%]	1.0% (0.0%, 1.9%)	
Chicago	[0%, 2%]	1.0% (0.1%, 2.0%)	
Dallas	[0%, 2%]	1.0% (0.1%, 2.0%)	
Houston	[0%, 2%]	0.9% (0.0%, 1.9%)	
Los Angeles	[0%, 2%]	1.0% (0.1%, 1.9%)	
Miami	[0%, 2%]	1.0% (0.0%, 1.9%)	
Minneapolis	[0%, 2%]	1.0% (0.1%, 1.9%)	
New York	[0%, 2%]	1.0% (0.1%, 2.0%)	
Philadelphia	[0%, 2%]	1.0% (0.0%, 1.9%)	
Phoenix	[0%, 2%]	1.0% (0.1%, 2.0%)	
Riverside	[0%, 2%]	1.0% (0.1%, 1.9%)	
San Diego	[0%, 2%]	1.0% (0.0%, 2.0%)	
San Francisco	[0%, 2%]	1.0% (0.1%, 1.9%)	
Seattle	[0%, 2%]	0.9% (0.1%, 1.9%)	
Washington	[0%, 2%]	1.0% (0.1%, 2.0%)	

# Mean and 95% percentile of parameter values that lead to simulated trajectories where the prevalence of resistance reaches at least 5% in the simulation.

‡ Since in our analysis, Drug B is introduced after Drug A, the prior distributions we chose for the initial prevalence of resistance to Drug A and B reflect the assumption that the prevalence of resistance to Drug B is initially smaller, on average, than the prevalence of resistance to Drug A.

Table F: Uncertainty range and feasible intervals for the annual importation rate of cases resistant to Drug A or Drug B in each metropolitan area

**Parameter**	**Uncertainty Range (All Uniform)**	**Mean and 95% Feasible Interval#**	**Sources to Inform Uncertainty Ranges**
Annual importation rate of cases resistant to Drug A or Drug B			Assumptions
Atlanta	[0, 5]	2.5 (0.1, 4.9)	
Boston	[0, 5]	2.4 (0.1, 4.9)	
Chicago	[0, 5]	2.5 (0.1, 4.9)	
Dallas	[0, 5]	2.5 (0.2, 4.9)	
Houston	[0, 5]	2.4 (0.1, 4.9)	
Los Angeles	[0, 5]	2.5 (0.1, 4.9)	
Miami	[0, 5]	2.5 (0.2, 4.8)	
Minneapolis	[0, 5]	2.6 (0.2, 4.9)	
New York	[0, 5]	2.6 (0.2, 4.9)	
Philadelphia	[0, 5]	2.7 (0.2, 4.9)	
Phoenix	[0, 5]	2.6 (0.2, 4.9)	
Riverside	[0, 5]	2.3 (0.2, 4.8)	
San Diego	[0, 5]	2.6 (0.3, 4.9)	
San Francisco	[0, 5]	2.4 (0.2, 4.9)	
Seattle	[0, 5]	2.5 (0.2, 4.9)	
Washington	[0, 5]	2.6 (0.1, 4.8)	

Mean and 95% percentile of parameter values that lead to simulated trajectories where the prevalence of resistance reaches at least 5% in the simulation.

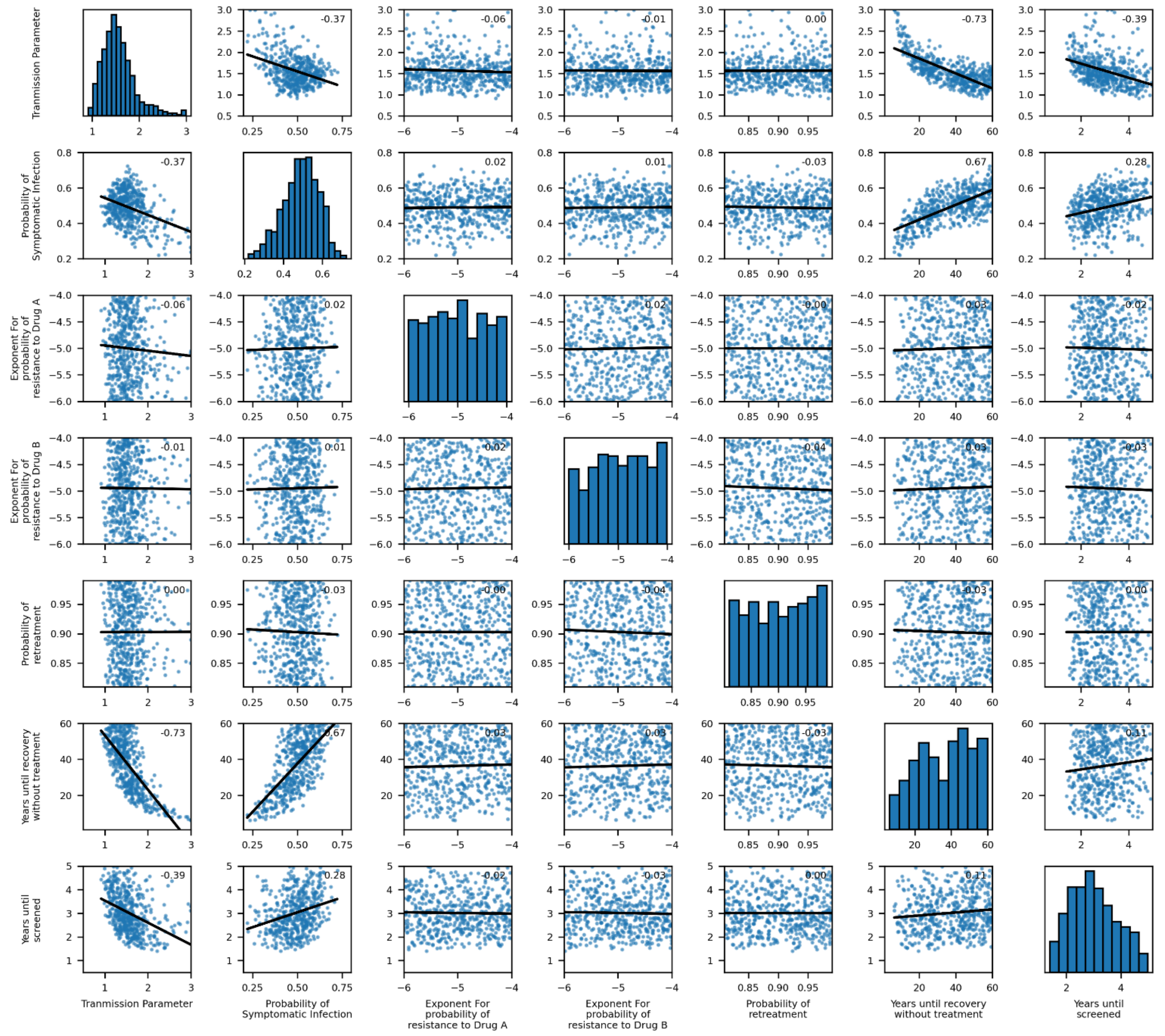

Fig G: Posterior distribution and the correlation between select key model parameters listed in Table B and Table C.

S4 Sensitivity Analyses

We performed the following analyses to evaluate the sensitivity of our conclusions to certain assumptions and the uncertainties in the model input parameters:

1. **Effect of parameters relating to resistance to Drug A or Drug B on the effective lifespan of Drugs A and B under the Base strategy**: To examine the effect of parameters relating to resistance to Drug A or Drug B on the effective lifespan of drugs A and B under the Base strategy, we calculated the Pearson's correlation, partial correlation, and partial rank correlation coefficients (which measures the monotonic relationship between an input parameter and the output after removing the linear effects of other parameters [1, 2]) for all parameters (Table I). The transmission parameter, duration of infection without treatment, time until screened for infection, the probability of retreatment after treatment failure, the exponent of the probability of developing resistance while receiving Drug A and Drug B, and the relative transmissibility of the strain resistance to Drug A and Drug B have an important effect on the effective lifespan of Drugs A and B under the Base strategy.
2. **Effect of parameters relating to resistance to Drug A or Drug B on the change in the effective lifespan of Drugs A and B**: To examine the effect of parameters relating to resistance to Drug A or Drug B on the performance of surveillance strategies (measured by the change in the effective lifespan of drugs A and B), we calculated the Pearson's correlation, partial correlation, and partial rank correlation coefficients for all parameters (Table J). Transmission parameter, duration of infection without treatment, and the relative transmissibility of the strain resistance to Drug A have an important effect on the change in the effective lifespan of Drugs A and B under the 'Spatial' strategy.
3. **Effect of simulation duration**: Fig H shows that our conclusions hold when we reduce the simulation duration from 50 years to 35 years.
4. **Effect of simulated trajectories used to calibrate the model**: To ensure that we have obtained enough simulated trajectories to calibrate our model, we repeated the calibration procedure described in §S3 using completely different parameter sets and random number seeds to simulate trajectories (in this analysis, the pseudo-likelihood for 5,000 trajectories was calculated and 231,993 trajectories were discarded dur to violating the feasibility conditions described in §S3.4). Projections under this analysis (Fig I) show that the comparative performance of the strategies considered is robust to the set of simulated trajectories used for calibrating our model.
5. **Effect of the choice of prior distributions and uncertainty ranges:** Fig G shows that the posterior distributions of the following parameters are bounded by their prior ranges: exponent for probability of resistance to Drug A, exponent for probability of resistance to Drug B, probability of retreatment, and years until recovery without treatment. To investigate if choosing a wider prior range for these parameters (and for other parameters that relate to the emergence and spread of resistance to Drugs A and B) would impact our conclusions, we repeated the calibration procedure described in §S3 using wider prior and

uncertainty ranges as listed in Table G-Table H. Projections under this analysis (Fig J) show that the comparative performance of the strategies considered is robust to the choice of prior distribution and uncertainty ranges used for calibrating our model.

6. **Effect of initial prevalence of resistance to Drug B:** In our main analysis, we assumed that, at time 0, the prevalence of resistance to Drug B is uniformly distributed over [0-2%]. This represent the scenario where Drug B is not solely used (or is a repurposed drug) for the treatment of gonorrhea. In this sensitivity analysis, we considered the situation where resistance to Drug B is initially zero representing the scenario where Drug B is a new antibiotic and used mainly to treat gonorrhea. Projections under this analysis (Fig K) show that the comparative performance of the strategies considered here is not sensitive to our assumption about the initial prevalence of resistance to Drug B.
7. **Effect of increase in transmission parameter**: The rate of reported gonorrhea cases among the MSM has increased significantly over the past few years. The estimated rate of gonorrhea cases increased 375.5% during 2010–2018 from 1,368.6 cases per 100,000 MSM in 2010 to 6,508.0 cases per 100,000 MSM in 2018 [14]. To evaluate the sensitivity of our conclusions to the increase in rate of gonorrhea cases, we considered a scenario where the value of the transmission parameter is increased by 10%. As displayed in Fig L, this scenario leads to simulation trajectories where the rate of gonorrhea cases increases over time. Our projections under this scenario (Fig M) show that our conclusions remain valid for the scenario where the rate of gonorrhea cases increases over time.

Table G: Prior distributions selected in the primary and sensitivity analyses.

Parameter	Prior Distribution (In Primary Analysis)	Prior Distribution (In Sensitivity Analysis)
Duration of infection (without treatment) (months)	[1, 60]	[1, 84]
Probability of retreatment after treatment failure with symptomatic infection	[80%, 100%]	[70%, 100%]

Table H: Uncertainty ranges selected in the primary and sensitivity analyses.

Parameter	Uncertainty Range (In Primary Analysis)	Uncertainty Range (In Sensitivity Analysis)
Probability of developing resistance while receiving Drug A	$10^{[-6, -4]}$	$10^{[-7, -3]}$
Probability of developing resistance while receiving Drug B	$10^{[-6, -4]}$	$10^{[-7, -3]}$
Initial prevalence of resistance to Drug A (one separate prior for each city, Table E)	[0%, 4%]	[0%, 6%]
Initial prevalence of resistance to Drug B (one separate prior for each city, Table E)	[0%, 2%]	[0%, 3%]
Annual importation rate of cases resistant to Drug A or Drug B (one separate prior for each city, Table F)	[0, 5]	[0, 7.5]
Relative transmissibility of the strain resistant to Drug A ($\gamma_A(t)$)		
b_A	[0, 0.2]	[0, 0.3]
Relative transmissibility of the strain resistant to Drug B or both drugs ($\gamma_B(t)$ and $\gamma_{AB}(t)$)		
b_B and b_{AB}	[0, 0.2]	[0, 0.3]

Table I: Correlation between select model input parameters and the effective lifespan of Drugs A and B under the 'Base' strategy.

	**Pearson's Correlation**		**Partial Correlation**		**Partial Rank Correlation**	
**Parameter**	**Coefficient**	***p*-value**	**Coefficient**	***p*-value**	**Coefficient**	***p*-value**
Transmission parameter (β) (1/year)	-0.0484	0.2801	0.0919	0.0399	0.1246	0.0053
Duration of infection (without treatment) (months)	-0.0356	0.4265	-0.1164	0.0092	-0.0882	0.0488
Time until screened for infection (years)	0.2482	0	0.6116	0	0.594	0
Time until seeking treatment for a symptomatic infection (days)	-0.024	0.5929	0.0095	0.8328	0.0116	0.7967
Time until retreatment (days)	0.0402	0.3693	-0.0219	0.6256	-0.0092	0.8372
Probability that an infection will be symptomatic	0.0032	0.9425	-0.1955	0	-0.2156	0
Probability of retreatment after treatment failure with symptomatic infection	0.0051	0.9086	0.0751	0.0934	0.0657	0.1422
Exponent of the probability of developing resistance while receiving Drug A	-0.0812	0.0698	-0.0317	0.48	-0.0605	0.177
Exponent of the probability of developing resistance while receiving Drug B	-0.0034	0.9399	0.0438	0.3288	0.0555	0.2154
Relative transmissibility of the strain resistant to Drug A ($\gamma_A(t)$)						
$b_{A,min}$	-0.3689	0	-0.6848	0	-0.7261	0
b_A	0.145	0.0011	-0.035	0.4346	-0.0578	0.1972
$t_{A,0}$	-0.0107	0.811	0.3972	0	0.4296	0
Relative transmissibility of the strain resistant to Drug B or both drugs ($\gamma_B(t)$ and $\gamma_{AB}(t)$)						
$b_{B,min}$ and $b_{AB,min}$	-0.5561	0	-0.7834	0	-0.8051	0
b_B and b_{AB}	0.1583	0.0004	-0.0062	0.8902	-0.0144	0.7473
$t_{B,0}$ and $t_{AB,0}$	0.2038	0	0.565	0	0.5978	0

Table J: Correlation between select model input parameters and the change in the effective lifespan of Drugs A and B under the 'Spatial' strategy with respect to the 'Base' strategy.

	**Pearson's Correlation**		**Partial Correlation**		**Partial Rank Correlation**	
**Parameter**	**Coefficient**	***p*-value**	**Coefficient**	***p*-value**	**Coefficient**	***p*-value**
Transmission parameter (β) (1/year)	0.3006	0	0.1181	0.0082	0.0568	0.2049
Duration of infection (without treatment) (months)	-0.2322	0	-0.0128	0.7753	-0.0395	0.3787
Time until screened for infection (years)	-0.2093	0	-0.1345	0.0026	-0.0984	0.0279
Time until seeking treatment for a symptomatic infection (days)	0.0437	0.3291	0.0236	0.5988	0.0308	0.4919
Time until retreatment (days)	0.0217	0.6284	0.0304	0.498	-0.0001	0.998
Probability that an infection will be symptomatic	-0.1557	0.0005	0.0046	0.9177	0.0203	0.6513
Probability of retreatment after treatment failure with symptomatic infection	-0.0596	0.1836	-0.0721	0.1071	-0.0855	0.056
Exponent of the probability of developing resistance while receiving Drug A	0.0737	0.0995	0.087	0.0518	0.0974	0.0295
Exponent of the probability of developing resistance while receiving Drug B	-0.0344	0.4428	-0.0231	0.6061	-0.0295	0.5108
Relative transmissibility of the strain resistant to Drug A ($\gamma_A(t)$)						
$b_{A,min}$	0.1358	0.0023	0.1942	0	0.2006	0
b_A	0.0174	0.6983	0.0395	0.3786	0.0366	0.4146
$t_{A,0}$	-0.0905	0.0432	-0.1758	0.0001	-0.1634	0.0002
Relative transmissibility of the strain resistant to Drug B or both drugs ($\gamma_B(t)$ and $\gamma_{AB}(t)$)						
$b_{B,min}$ and $b_{AB,min}$	0.0086	0.8476	-0.002	0.9639	-0.0125	0.7802
b_B and b_{AB}	-0.1093	0.0144	-0.1102	0.0137	-0.1085	0.0152
$t_{B,0}$ and $t_{AB,0}$	-0.067	0.1349	-0.0699	0.1187	-0.0732	0.1022

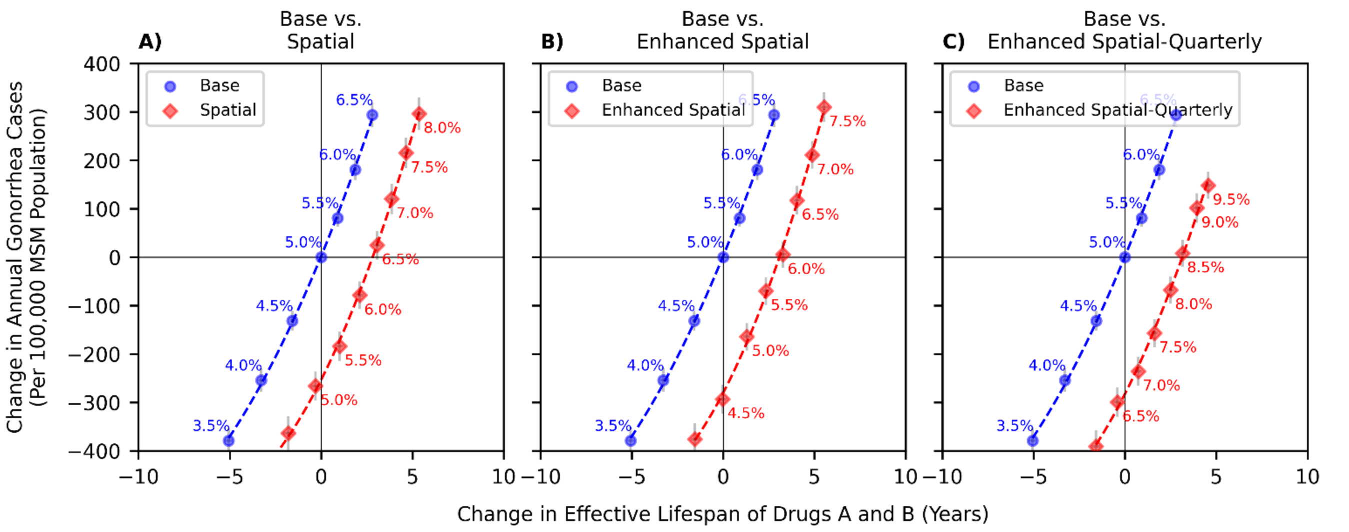

Fig H: Comparing the performance of policies in Table 1 with respect to the current policy over a 35-year simulation window. The bars represent 95% confidence intervals.

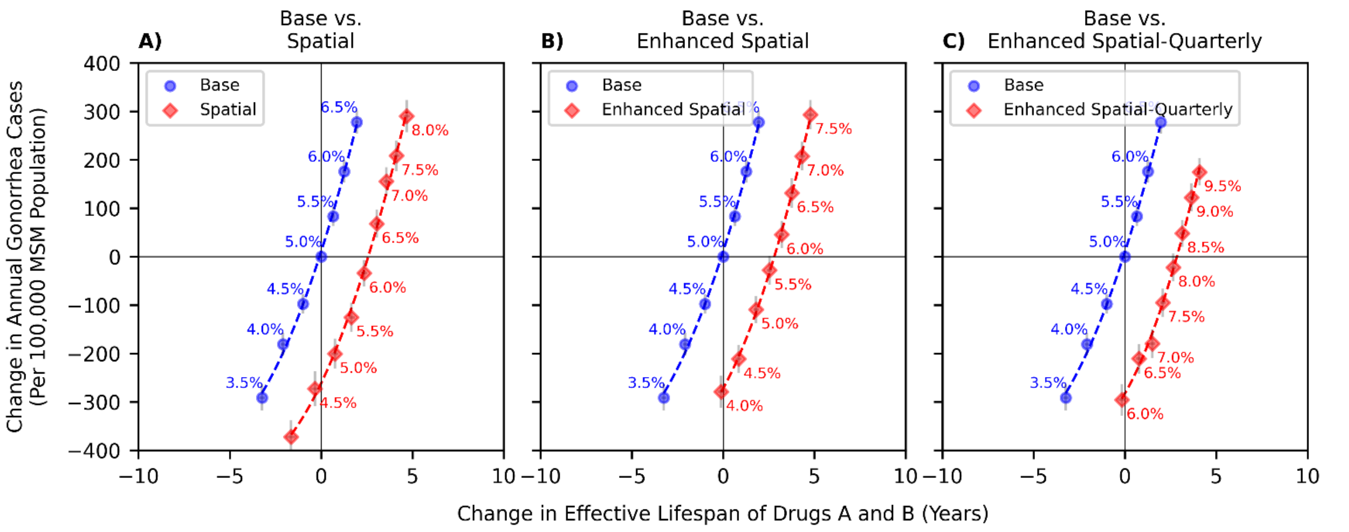

Fig I: Comparing the performance of policies in Table 1 with respect to the current policy using the recalibrated model. The bars represent 95% confidence intervals.

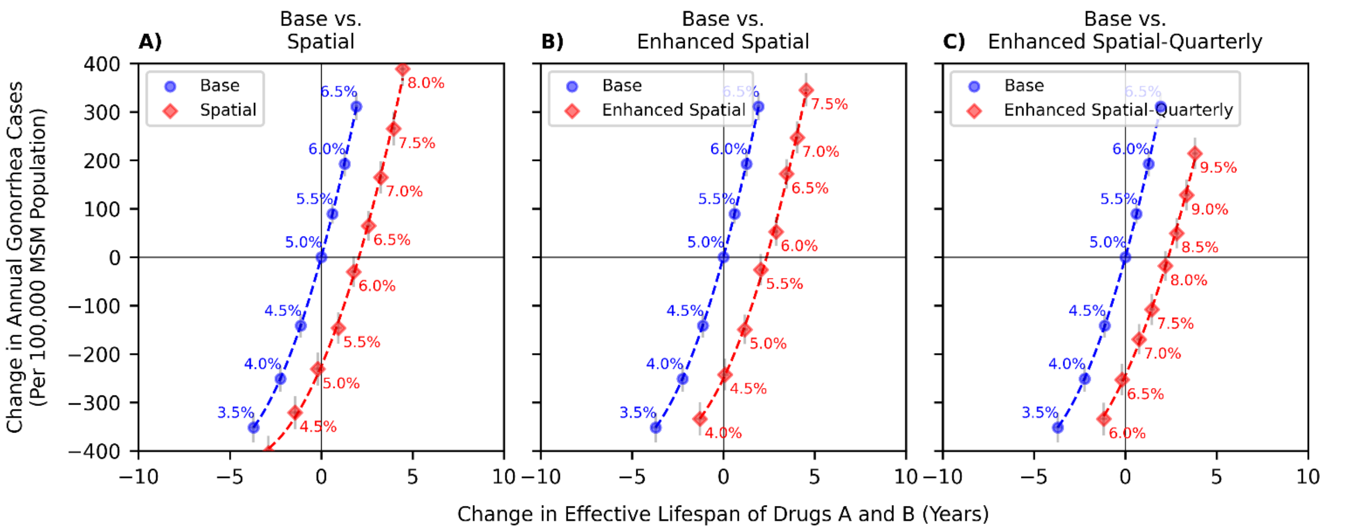

Fig J: Comparing the performance of policies in Table 1 with respect to the current policy using the recalibrated model where wider prior distributions and uncertainty ranges are selected (Table G-Table H). The bars represent 95% confidence intervals.

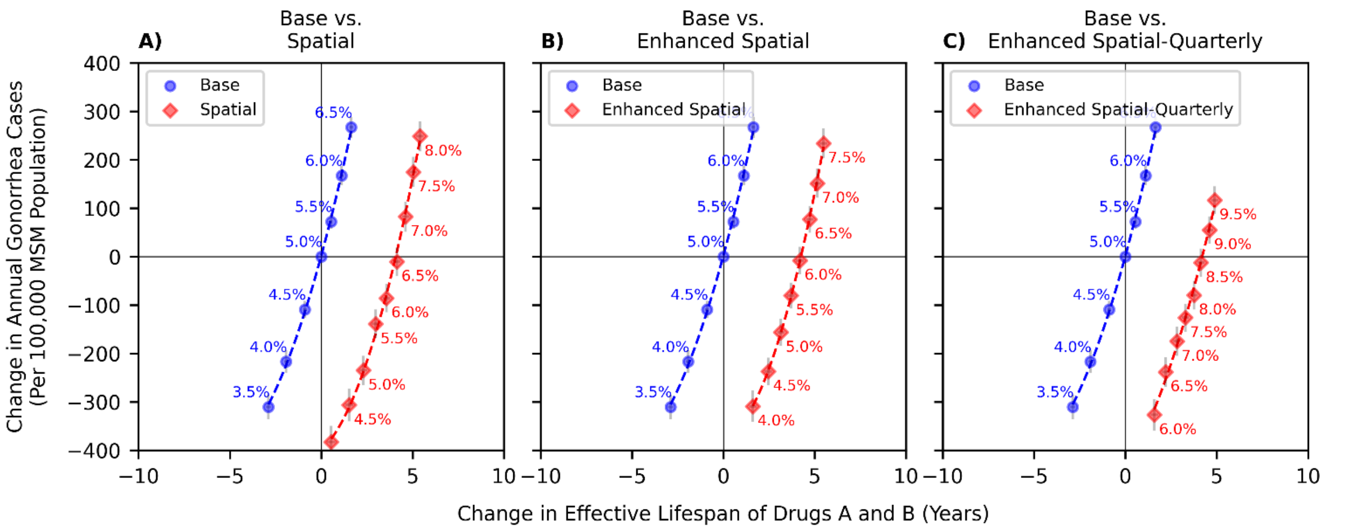

Fig K: Comparing the performance of policies in Table 1 with respect to the current policy using when the prevalence of resistance to Drug B is zero. The bars represent 95% confidence intervals.

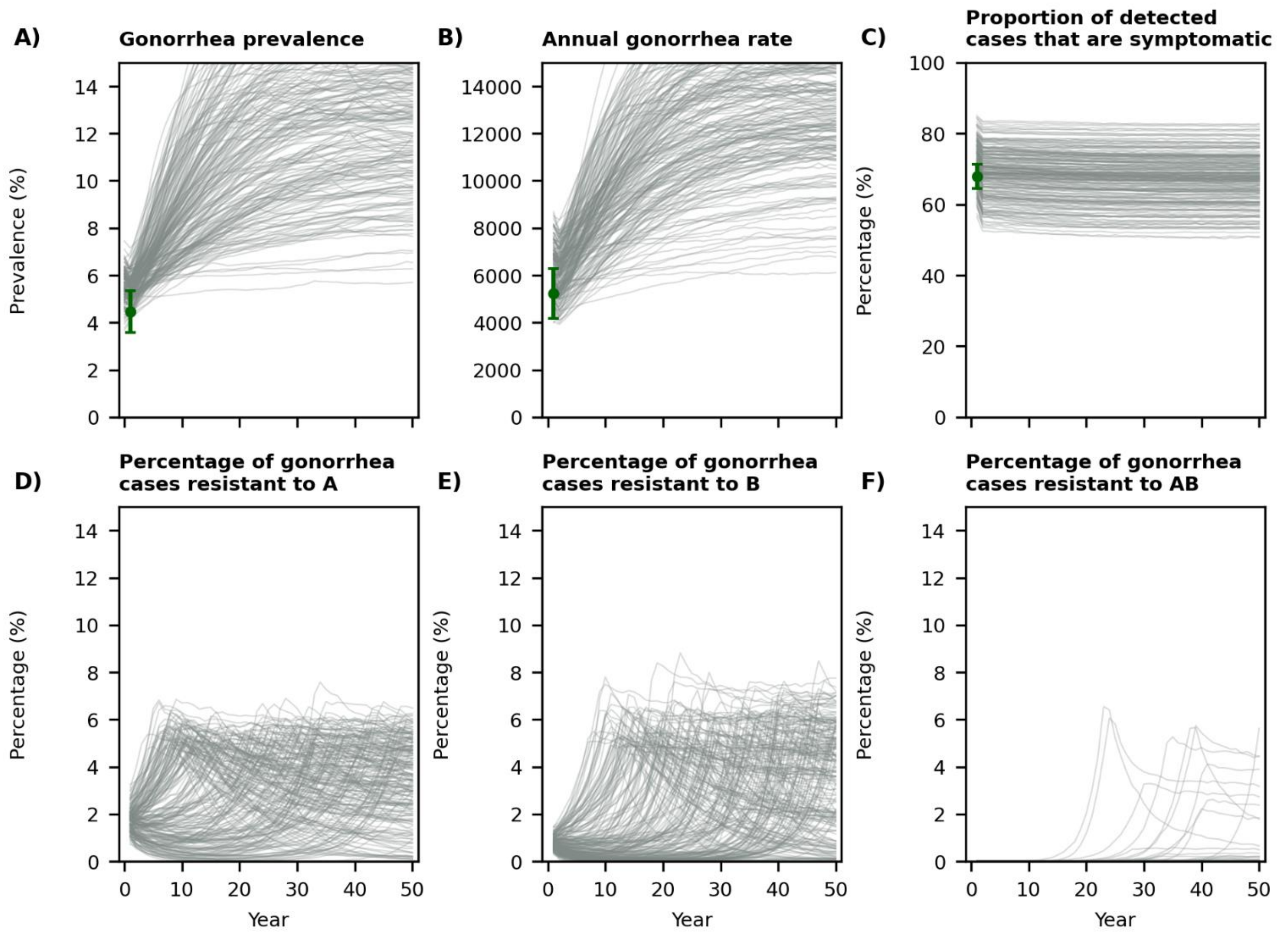

Fig L: Displaying 200 simulated trajectories from the calibrated model where the value of transmission parameter is increased by 10%.

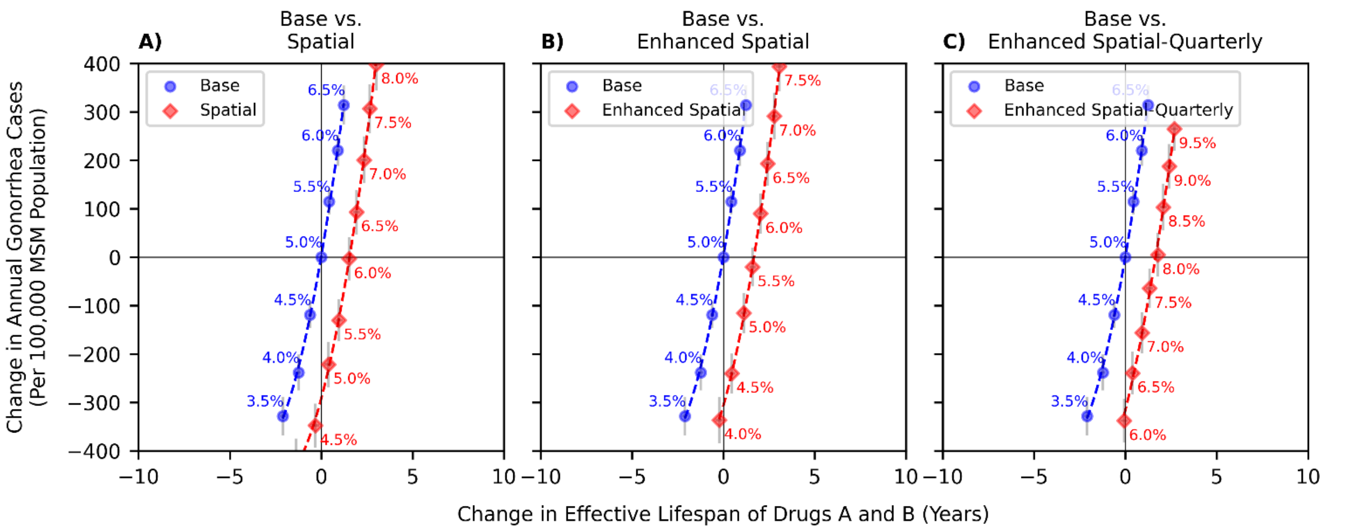

Fig M: Comparing the performance of policies in Table 1 with respect to the current policy using when the value of the transmission parameter is increased by 10% (Fig L). The bars represent 95% confidence intervals.

References

1. Grey JA, Bernstein KT, Sullivan PS, Purcell DW, Chesson HW, Gift TL, et al. Estimating the Population Sizes of Men Who Have Sex With Men in US States and Counties Using Data From the American Community Survey. JMIR Public Health Surveill. 2016;2(1):e14. Epub 2016/05/27. doi: 10.2196/publichealth.5365. PubMed PMID: 27227149; PubMed Central PMCID: PMCPMC4873305.
2. Centers for Disease Control and Prevention. Sexually Transmitted Disease Surveillance 2017. Atlanta, U.S.: U.S. Department of Health and Human Services, 2018 https://www.cdc.gov/std/stats17/2017-STD-Surveillance-Report_CDC-clearance-9.10.18.pdf.
3. Stenger MR, Pathela P, Anschuetz G, Bauer H, Simon J, Kohn R, et al. Increases in the Rate of Neisseria gonorrhoeae Among Gay, Bisexual and Other Men Who Have Sex With Men-Findings From the Sexually Transmitted Disease Surveillance Network 2010-2015. Sex Transm Dis. 2017;44(7):393-7. Epub 2017/06/14. doi: 10.1097/OLQ.0000000000000623. PubMed PMID: 28608788; PubMed Central PMCID: PMCPMC5508584.
4. Yaesoubi R, Cohen T, Hsu K, Gift TL, Chesson H, Salomon JA, et al. Adaptive guidelines for the treatment of gonorrhea to increase the effective life span of antibiotics among men who have sex with men in the United States: A mathematical modeling study. PLoS Med. 2020;17(4):e1003077. doi: 10.1371/journal.pmed.1003077. PubMed PMID: 32243443; PubMed Central PMCID: PMC7122693 following competing interests: RY, TC, JAS, and YHG received funding from the National Institute of Health and the US Centers for Disease Control and Prevention. KH is employed by Massachusetts Department of Public Health. TLG and HC are employed by the US Centers for Disease Control and Prevention.
5. Kirkcaldy RD, Harvey A, Papp JR, Del Rio C, Soge OO, Holmes KK, et al. *Neisseria gonorrhoeae* Antimicrobial Susceptibility Surveillance - The Gonococcal Isolate Surveillance Project, 27 Sites, United States, 2014. MMWR Surveill Summ. 2016;65(7):1-19. Epub 2016/07/16. doi: 10.15585/mmwr.ss6507a1. PubMed PMID: 27414503.
6. Johnson Jones ML, Chapin-Bardales J, Bizune D, Papp JR, Phillips C, Kirkcaldy RD, et al. Extragenital Chlamydia and Gonorrhea Among Community Venue-Attending Men Who Have Sex with Men - Five Cities, United States, 2017. MMWR Morb Mortal Wkly Rep. 2019;68(14):321-5. doi: 10.15585/mmwr.mm6814a1. PubMed PMID: 30973847; PubMed Central PMCID: PMC6459584 potential conflicts of interest. No potential conflicts of interest were disclosed.
7. Newman LM, Dowell D, Bernstein K, Donnelly J, Martins S, Stenger M, et al. A tale of two gonorrhea epidemics: results from the STD surveillance network. Public Health Rep. 2012;127(3):282-92. Epub 2012/05/02. doi: 10.1177/003335491212700308. PubMed PMID: 22547859; PubMed Central PMCID: PMCPMC3314072.
8. Murphy DR, Klein RW, Smolen LJ, Klein TM, Roberts SD. Using Common Random Numbers in Health Care Cost-Effectiveness Simulation Modeling. Health services research. 2013;48(4):1508-25.
9. Whittles LK, White PJ, Didelot X. Estimating the fitness cost and benefit of cefixime resistance in Neisseria gonorrhoeae to inform prescription policy: A modelling study. PLoS Med. 2017;14(10):e1002416. Epub 2017/11/01. doi: 10.1371/journal.pmed.1002416. PubMed PMID: 29088226; PubMed Central PMCID: PMCPMC5663337.
10. Tuite AR, Gift TL, Chesson HW, Hsu K, Salomon JA, Grad YH. Impact of Rapid Susceptibility Testing and Antibiotic Selection Strategy on the Emergence and Spread of Antibiotic Resistance in Gonorrhea. J Infect Dis. 2017;216(9):1141-9. Epub 2017/10/03. doi: 10.1093/infdis/jix450. PubMed PMID: 28968710; PubMed Central PMCID: PMCPMC5853443.
11. Garnett GP, Mertz KJ, Finelli L, Levine WC, St Louis ME. The transmission dynamics of gonorrhoea: modelling the reported behaviour of infected patients from Newark, New Jersey. Philos Trans R Soc Lond B Biol Sci. 1999;354(1384):787-97. Epub 1999/06/12. doi: 10.1098/rstb.1999.0431. PubMed PMID: 10365404; PubMed Central PMCID: PMCPMC1692556.
12. Hui BB, Whiley DM, Donovan B, Law MG, Regan DG, Investigators GS. Identifying factors that lead to the persistence of imported gonorrhoeae strains: a modelling study. Sex Transm Infect. 2017;93(3):221-5. Epub 2017/04/23. doi: 10.1136/sextrans-2016-052738. PubMed PMID: 28432206.

13. Kirkcaldy RD, Hook EW, 3rd, Soge OO, del Rio C, Kubin G, Zenilman JM, et al. Trends in Neisseria gonorrhoeae Susceptibility to Cephalosporins in the United States, 2006-2014. JAMA. 2015;314(17):1869-71. Epub 2015/11/04. doi: 10.1001/jama.2015.10347. PubMed PMID: 26529166; PubMed Central PMCID: PMCPMC4788090.
14. Centers for Disease Control and Prevention. Sexually Transmitted Disease Surveillance 2018. Atlanta, U.S.: U.S. Department of Health and Human Services, 2019 https://www.cdc.gov/std/stats18/STDSurveillance2018-full-report.pdf.
