# Supplementary material for: Evaluating spatially adaptive guidelines for the treatment of gonorrhea to reduce the incidence of gonococcal infection and increase the effective lifespan of antibiotics
Source: PLoS Comput Biol. 2022 Feb 9;18(2):e1009842. doi: 10.1371/journal.pcbi.1009842 (PMC8863219; doi:10.1371/journal.pcbi.1009842)
Supplement: Attachment — Submitted filename: Response to Reviewers.pdf [file pcbi.1009842.s002.pdf]

Responses to Reviewer 1

"This paper assesses whether localised gonorrhea treatment guidelines based on regional estimates of resistance prevalence could be useful in combating the development of antibiotic resistance and thereby extending the clinical lifespan of antibiotics. Overall the paper is a timely and well written modelling analysis on a topic of great importance, namely: preserving the utility of antibiotics against Neisseria gonorrhoeae.

The authors adapt a previously-published individual-based model, which they fit to the US gonorrhea epidemic in men who have sex with men, calibrating to regional and national incidence, national prevalence and the proportion of cases presenting with symptoms in 2017. The model is clearly described and well-documented in the supplementary material. The authors use their calibrated model to perform simulation analyses to assess the impact of regional treatment guidelines on both the burden of gonorrhea and the lifespan of gonorrhea treatments (based on the development of antibiotic resistance).

The idea of regional treatment guidelines is novel and important, and the methodology of the analysis is sound. However, I have some concerns about implicit assumptions in the model calibration, namely the assumption of endemic equilibrium prevalence and incidence given the current rapidly increasing epidemic of gonorrhea. Further, some of the claims in the abstract, particularly around the lifespan extension achieved, are overstated as they rely on increasing the threshold of resistance at which drugs are abandoned (from 5% to e.g. 6.5%) which seems unlikely to be implemented in practice.

I have detailed my comments and suggestions below."

Model Calibration:

(1) "The authors calibrate to constant incidence over a period of 10 years (at least this is what the equations for L2 and L3 seem to suggest – I may have misunderstood) based on 2017 data assuming 5,548 cases per 100,000. Are these 10 years 2007 – 2017? Because over that time period gonorrhea incidence has increased by around 90%. Do the authors' conclusions still hold in an increasing epidemic?"

Response: Thank you for raising this important point. As indicated by the Reviewer, the rate of reported gonorrhea cases among the MSM population has increased significantly over the past few years. The estimated gonorrhea case rate increased 375.5% during 2010–2018 from 1,368.6 cases per 100,000 MSM in 2010 to 6,508.0 cases per 100,000 MSM in 2018 (according to the CDC's Sexually Transmitted Disease Surveillance 2018 report). We sought to address the reviewer's comment by evaluating the sensitivity of our conclusions to increasing prevalence in the MSM population by considering a scenario in which the value of the transmission parameter is increased by 10% (a change which could be understood to reflect decreased use of condoms, for example). As displayed in the figure below (Fig. S10), this scenario leads to simulation trajectories where the rate of gonorrhea cases increases over time but reaches a new equilibrium after a few years. Our projections under this scenario (Fig. S11) show that our conclusions remain valid for the scenario where the rate of gonorrhea cases increases over time.

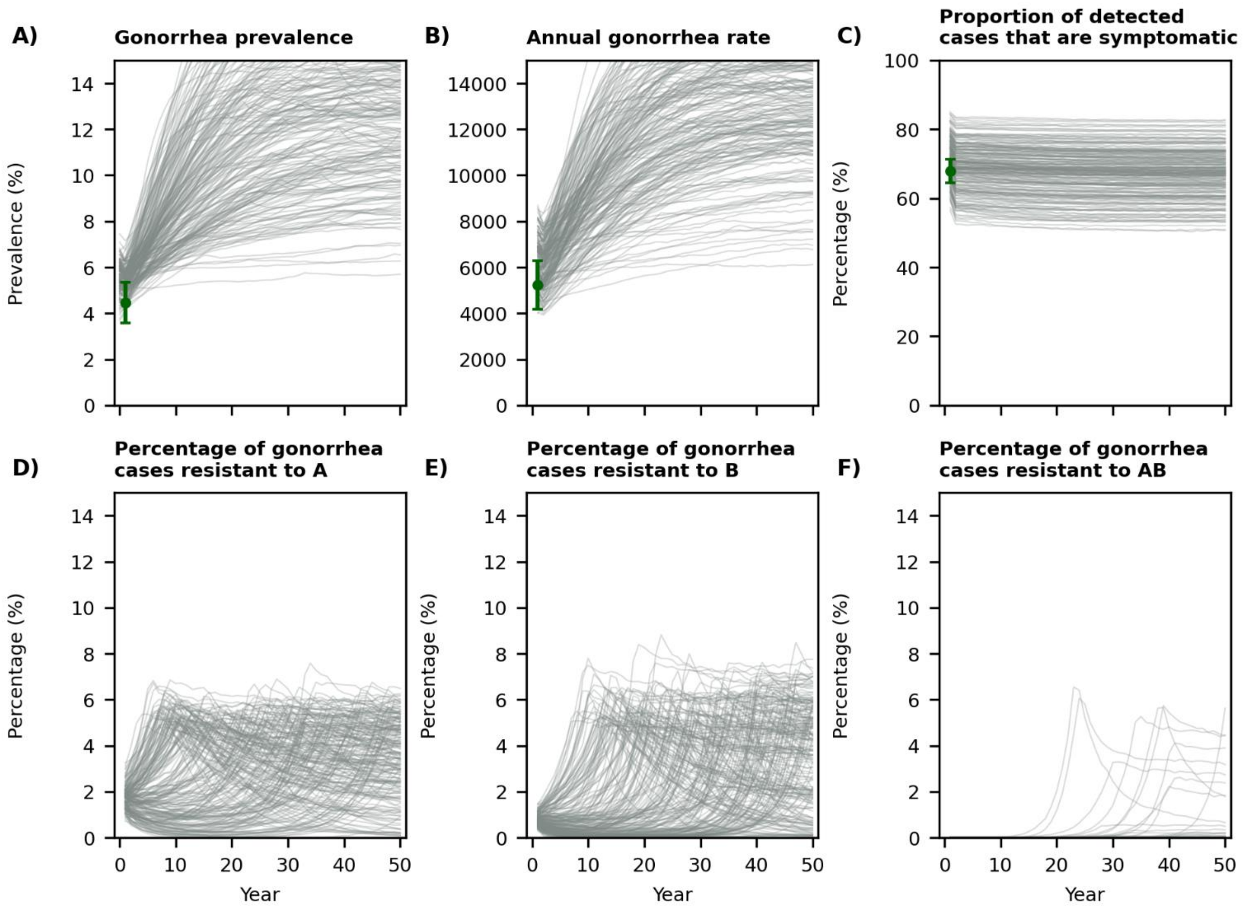

Fig. S10: Displaying 200 simulated trajectories from the calibrated model where the value of transmission parameter is increased by 10%.

We have added the following text to the section "S4. Sensitivity Analysis":

> "**7. Effect of increase in transmission parameter**: The rate of reported gonorrhea cases among the MSM population has increased significantly over the past few years. The estimated rate of gonorrhea cases increased 375.5% during 2010–2018 from 1,368.6 cases per 100,000 MSM in 2010 to 6,508.0 cases per 100,000 MSM in 2018 [14]. To evaluate the sensitivity of our conclusions to the increase in rate of gonorrhea cases, we considered a scenario where the value of the transmission parameter is increased by 10%. As displayed in Fig. S10, this scenario leads to simulation trajectories where the rate of gonorrhea cases increases over time. Our projections under this scenario (Fig. S11) show that our conclusions remain valid for the scenario where the rate of gonorrhea cases increases over time"

We also added the following text to the end of the Result section:

> "While the rate of reported gonorrhea cases among the MSM population has increased significantly over the past few years [14], our model was calibrated to a constant incidence and prevalence (Fig. 2A-B). To evaluate the sensitivity of our conclusions to the increase in rate of gonorrhea cases, we also modelled a scenario where the value of the transmission parameter is increased by 10%, which led to simulation trajectories with increasing rate of gonorrhea cases and the prevalence of infection (Fig.

S10). Our projections show that our conclusions remain valid under this scenario (Fig. S11). The results of other sensitivity analyses we conducted are presented in §S4 of the Supplement."

(2) "The authors calibrate to a prevalence of 4.5% (3.6%-5.4%) in 2017 for the whole USA (L1). The reference used to support this assumption (Johnson Jones ML, Chapin-Bardales J, Bizune D, Papp JR, Phillips C, Kirkcaldy RD, et al. Extragenital Chlamydia and Gonorrhea Among Community Venue-Attending Men Who Have Sex with Men - Five Cities) includes a breakdown of prevalence by city, all of which are locations modelled in the manuscript, which suggests prevalence varies substantially by location: e.g. 1.8% (0.5% - 3.1%) San Francisco vs 6.7% (4.4% - 9.0%) in Houston. Is it possible to fit to these prevalence estimates in the locations presented? I note that this will likely be hard to achieve, since the prevalence and incidence estimates may be irreconcilable: in Table S1 San Francisco has much higher incidence than Houston (7,931 cases per 100,000 vs 4,858)."

Response: Thank you for this suggestion. We recalibrated the model using estimates for the prevalence of gonorrhea in Houston, Miami, New York, San Francisco, and Washington, DC (obtained from Johnson Jones et al., *MMWR Morb Mortal Wkly Rep*. 2019;68(14):321-325) as new calibration targets. We have revised the section "S3. Model calibration" and have added a new figure in the supplement (Fig. S3) to show the fit of our model to these new calibration targets. All results are updated accordingly.

(3) "In calculating their 2017 regional incidence estimates for model calibration (Table S1), the authors assume that the proportion of all gonorrhea cases attributable to MSM is the same in each location– is there any evidence to support the validity of this assumption? The fact that the inferred incidence seems to contradict the prevalence reported by Johnson Jones et. al, as noted above, suggests there may be underlying heterogeneity."

Response: Thank you for pointing this out. We found estimates for the reported number of gonorrhea cases among the MSM population in New York City, Philadelphia, and San Francisco provided by Stenger MR et al. (*Sex Transm Dis*. 2017;44(7):393-397). We, however, were not able to find any reliable estimate for the reported rate of gonorrhea cases or any estimate for the percentage of gonorrhea cases that occurred among the MSM population of other cities. Nevertheless, we note that we now calculate the reported number of gonorrhea cases among the MSM population of cities considered here at 5,285 per 100,000 MSM (Table S1), which is consistent with the estimate of 5,241.8 per 100,000 MSM provided by the CDC's Sexually Transmitted Disease Surveillance 2018 report. Table S1 and all results are updated accordingly. We now discuss this as a limitation of our study in the Discussion section:

> "Third, we found estimates for the reported number of gonorrhea cases among the MSM population of only 3 of the 16 cities included in our model (New York City, Philadelphia, and San Francisco) [22]. For the remaining 13 cities, we assumed that the proportion of all gonorrhea cases attributable to MSM is the same for each location (Table S1). Despite this assumption, we note that the calculated number of reported gonorrhea cases among the MSM population of cities considered here (i.e., 5,285 per 100,000 MSM, Table S1) is consistent with the estimate of 5,241.8 per 100,000 MSM provided by the CDC's Sexually Transmitted Disease Surveillance 2018 report [1]."

Simulation study

(4) "Methods pg3: Using a t-distribution to calculate binomial proportion confidence intervals is not best practice due to the possibility of the bounds overshooting the unit interval. It would be better to use Wilson's method which results in a 95% CI for estimating 5% prevalence is 2.7%-9.0% for a sample of 200 (vs 2.0%-8.0%) and 3.7%-6.7% for a sample of 800 (vs 3.5%-6.5%)."

Response: Thank you for this suggestion. We revised the text accordingly:

> "With 200 susceptibility tests, the local prevalence of resistance can be estimated with a 95% Wilson's confidence interval of 2.7%-9.0% if the true prevalence of resistance reaches 5%. This confidence interval can be reduced to 3.7%-6.7% when 800 susceptibility tests are performed."

(5) "Measure of number of additional cases treated is essentially averaged over whole duration of simulation (i.e. incidence is perfectly flat)"

Response: We appreciate the opportunity to clarify this point. As displayed in Figure 2B, we do not assume that the incidence is flat. To estimate the expected number of additional cases treated under a given strategy, we first estimate the number of years that the effective lifespan of Drugs A and B can be extended under the strategy (ΔL) (this is essentially the distance between the blue and red curves in Fig. 4 where they intercept the x-axis). We then approximate the expected number of additional cases of gonorrhea that would be treated successfully with first-line antibiotics under this strategy during the simulation period with $S_0\frac{\Delta L}{L_0}$, where S_0 is the total number of cases successfully treated with Drugs A or B during the simulation period, and L_0 is the effective lifespan of Drugs A and B under the status quo.

(6) "The authors' conclusion that the lifespan of a drug could be extended by 2.71 years rely on moving the resistance threshold as well as switching to a new prescribing strategy (Fig 4A), however while discussed this aspect is not emphasised, particularly in the abstract. Is it likely that the resistance threshold would be increased in practice? The policy would be very difficult to communicate and seems to tacitly assume that maintaining the current level of gonorrhea as a key goal. This is problematic in itself as the authors' simulations assume the epidemic of gonorrhea is at equilibrium (i.e. stable) whereas gonorrhea rates have been increasing by around 5% each year in US MSM. The more realistic interpretation of the work is that the policies considered could modestly extend the lifespan of the drugs (< 1yr), but that their main impact would be reducing the annual number of cases and the burden of gonorrhea (if the current 5% threshold were kept in place), and that the lifespan could be increased further, but that this would require a reformulation of the threshold rule."

Response: Thank you for this great suggestion. We have made revisions throughout the manuscript to reflect this interpretation. For example, the Finding section of the Abstract now reads:

> "Under the current strategy of updating the standardized treatment guideline when the prevalence of resistance passes the 5% threshold, we showed that spatially adaptive guidelines could reduce the annual rate of gonorrhea cases by 200 cases (95% uncertainty interval: 169, 232) per 100,000 MSM population while extending the use of a first-line antibiotic by 0.75 (0.55, 0.95) years."

We take a similar approach to present our findings in the Result section, in addition to reporting the maximum increase in the effective lifespan of antibiotics if the resistance threshold is changed.

(7) Minor points:

a) "Fig1 is an exact replica of Fig1 in Yaesoubi2020, re-publishing the figure improves the readability of the paper, but I recommend acknowledging the fact and citing in the caption."

<u>Response</u>: We have included the following text in the caption of Fig 1:

> "(adapted from [15] and extended to simulate the transmission of N. gonorrhoeae among men who have sex with men in 16 metropolitan areas in the United States)."

b) "Pg9: Operationalizing -> Implementing"

<u>Response</u>: We replaced "Operationalizing" with "Implementing".

c) "Pg9: Therefore, for regions without a surveillance site, it would need to be decided that data from which surveillance site(s) should be used to inform treatment guidelines"

<u>Response</u>: We have deleted this confusing sentence and re-written the prior sentence to read: "First, actionable surveillance of AMR gonorrhea may not be conducted in every geographic region, thereby requiring a policy for how best to inform guidelines for regions lacking surveillance."

d) "Pg9: optimality of the number –> the optimal number"

<u>Response</u>: We revised as suggested.

e) "S3 pg5: replies -> relies"

<u>Response</u>: Thank you. We corrected this typo.

Responses to Reviewer 2

"The authors use a mathematical model of the spread of gonorrhea to explore whether locally determined rather than national empiric guidelines would increase the lifespan of antibiotics."

(1) "It was unclear to me whether such a complex model was needed to answer this question. Moreover, it was unclear whether the results (5% more could be treated, 284/5548) were really that supportive of the strategy which presumably comes with substantial local surveillance and guideline formation costs."

Response: Thank you for raising these important points.

1. We have strived to keep our model parsimonious, including only details essential to address our study's questions quantitatively. As displayed in Fig 1 and discussed in section "Simulating guidelines for the first-line treatment of gonorrhea", our model seeks to do so by assessing spread of AMR gonorrhea in multiple metropolitan areas; by distinguishing between symptomatic and asymptomatic cases as these may contribute differently to the spread of gonorrhea; and by incorporating susceptibility to first-line antibiotic treatment. However, we recognize that there may well be other approaches to the study's questions, and we would very much welcome studies that employ other models and strategies.
2. We agree with the Reviewer that the impact of spatially adaptive treatment guidelines on the effective lifespan of existing antibiotics are relatively small. As per Reviewer 1's comment (#6 above), we note that these strategies could have a more substantial impact on the rate of gonorrhea cases (see also Fig 4).
3. We agree with the Reviewer that enhancing surveillance systems could be costly and the value of the proposed strategies should be investigated through cost-effectiveness analyses. However, arriving at a conclusion regarding the costs requires an estimate of the potential benefits, as we have endeavored to do in this project. We have updated the Discussion to include this point:

    > "Finally, enhancing surveillance systems to expand the geographic location of surveillance sites and to enable more frequent reporting and evaluation of more gonococcal isolates would increase the cost of surveillance. Therefore, the value of strategies proposed here should be investigated through cost-effectiveness analyses."

(2) "Abstract: An aim is to explore the spread of drug-resistant gonorrhea, but there is no conclusion related to this."

Response: Thank you for pointing this out. We revised the Background section of the Abstract:

> "In the absence of point-of-care gonorrhea diagnostics that report antibiotic susceptibility, gonorrhea treatment is empiric and determined by standardized guidelines. These guidelines are informed by estimates of resistance prevalence from national surveillance systems. <u>We examined whether locally-informed guidelines could reduce the incidence of gonorrhea and increase the effective lifespan of antibiotics used in standardized treatment guidelines</u>."

(3) "Introduction: How long does it usually take to get DSTs? How long is the empiric prescribing time? Isn't there an issue with such sexually transmitted pathogens of losing a patient – so how often does a switch actually happen?"

Response: DST is rarely done; when it is performed, it takes several days to obtain results. Treatment is most commonly through administration of a single dose of antibiotics. Therefore, gonorrhea treatment is empiric and informed by guidelines. These guidelines are revisited when the prevalence of resistance increases, with shifts usually associated when resistance exceeds a 5% threshold. These changes have occurred regularly (Figure 5 in Quillin SJ, Seifert HS. *Nat Rev Microbiol*. 2018;16(4):226-240), including changing from quinolones to cephalosporins in 2007, removing cefixime from recommended regimens in 2012, restricting to dual therapy with ceftriaxone and azithromycin in 2015, and to ceftriaxone alone this past year.

(4) "Methods: Resistance to the no longer used drugs may increase due to bystander selection not necessarily due to a decreased fitness cost."

Response: We agree with the Reviewer that increasing bystander selection is one mechanism that could lead to increased prevalence of *N. gonorrhoeae* resistance to antibiotics no longer recommended for first-line treatment; this is an interesting hypothesis and worth investigation. For the modeling here, we opted to focus on decreasing fitness cost from resistance mutations because of precedent and because of evidence in support of this phenomenon (Andersson DI and Hughes D. *Nat Rev Microbiol.* 2010;8(4):260-271; Schulz zur Wiesch P et al. *Antimicrob Agents Chemother* 2010; 54:2085–95; and Tuite AR et al.. *J Infect Dis*. 2017;216(9):1141-1149).

(5) "Figure 3: "This figure demonstrates that our simulation model can describe the geographical variation in the trends of ciprofloxacin resistant gonorrhea observed in GISP." I'm not sure that this figure does show this."

Response: Thank you for pointing this out. We sought to clarify the caption of Fig. 3 as follows:

> "This figure demonstrates that the rate at which resistance to Drug A increases within the 16 metropolitan areas of our model is consistent with the geographical variation in the trends of ciprofloxacin-resistant gonorrhea observed in GISP."
