# Supplementary material for: Evaluating spatially adaptive guidelines for the treatment of gonorrhea to reduce the incidence of gonococcal infection and increase the effective lifespan of antibiotics
Source: PLoS Comput Biol. 2022 Feb 9;18(2):e1009842. doi: 10.1371/journal.pcbi.1009842 (PMC8863219; doi:10.1371/journal.pcbi.1009842)
Supplement: Attachment — Submitted filename: Response to Reviewers.pdf [file pcbi.1009842.s003.pdf]

Responses to Reviewer 3

"The authors investigate how spatially informed guidelines for gonorrhea treatment can improve the lifespan of antibiotics and the number of gonorrhea cases. To this end, they propose an individual-based epidemiological and evolutionary model describing the incidence of gonorrhea in several American cities. They describe symptomatic vs. asymptomatic infections, several susceptible and resistant gonorrhea genotypes, and treatment by three possible antibiotics (A, B, last-line). They derive plausible sets of parameters reproducing well the overall prevalence & yearly incidence of gonorrhea in the USA, fraction symptomatic, and for which drug resistance for both A and B reaches at least 5% over the 50 years of the simulation.

The manuscript is interesting and although the benefits of a spatially informed strategy appear weak, it is still a worthwhile exploration.

My major comments are the following:"

(1) "Clarity of the model description can be improved. Specific suggestions:"

a) "Clarify the structure of treatment, particularly under what circumstances individuals move to drug A, drug B, drug M, and what happens in case of treatment success vs. failure. Figure 1 is not very informative in this respect. I don't find the diagram of fig. S1 very clear in terms of the type of treatment applied depending on stage, resistance status, symptoms. (e.g. Asymptomatic can seek second line treatment?). Please improve this. Paragraph S1.2 need to specify in more details what happens to individuals on adequate / inadequate treatment."

Response: Thank you for raising this concern. We revised our description of the model in the main text and the Supplement to clarify our assumptions related to treatment and treatment outcomes. In the main text, we added the following to the Methods section:

> "Infected individuals who seek treatment or are detected through screening will receive treatment with either Drug A, B, or M, depending on the current recommendation for first-line therapy. We assume that treatment always fails if the infecting strain is resistant to the prescribed antibiotic. Treatment with an antibiotic to which the infecting strain is susceptible may lead to one of two possible outcomes: 1) the treatment succeeds and the individual returns to the susceptible state, or 2) the treatment leads to resistance and fails (Fig. S1). A fraction of symptomatic individuals failing the first-line treatment (due to receiving ineffective treatment or developing resistance) will seek retreatment with some delay. These individuals will receive a second-line antibiotic, which is Drug B in the absence of resistance to Drug B, and Drug M in the case of resistance to Drug B. We assume that treatment with Drug B could lead to the selection of resistance to Drug B with a small probability, upon which the individual will be treat with Drug M (Fig. S1). We further assume that treatment with Drug M is always successful. As soon as effective treatment (i.e., treatment with an antibiotic that matches the individual's susceptibility profile) is initiated, we assume that infected individuals no longer contribute to the force of infection (due to either negligible infectiousness or reduced sexual activity). Those symptomatic individuals who fail first-line treatment but do not seek retreatment will remain in their current state where they may naturally recover or seek treatment in future time steps. Asymptomatic individuals may be detected only through screening."

In §S1.2 of the Supplement, we now provide the list of assumptions related to treatment and treatment outcomes:

- "Asymptomatic cases do not seek treatment and remain infectious until they recover spontaneously or get detected through active screening.
- Symptomatic cases who seek treatment and asymptomatic cases who are detected through screening will receive treatment with either Drug A, B, or M, depending on the current recommendation for first-line therapy.
- Treatment always fails if the infecting strain is resistant to the prescribed antibiotic.
- Treatment with an antibiotic to which the infecting strain is susceptible may lead to one of two possible outcomes: 1) the treatment succeeds and the individual returns to the susceptible state, or 2) the treatment leads to resistance and fails.
    - A fraction of symptomatic individuals failing the first-line treatment (due to receiving ineffective treatment or developing resistance) will seek retreatment with some delay. These individuals will receive a second-line antibiotic which is Drug B in case of resistance to Drug A, and is Drug M in case of resistance to Drug B.
    - We assume that treatment with Drug M is always successful but treatment with Drug B could lead to the selection of resistance with a small probability, upon which the individual will be treat with Drug M
    - Those symptomatic individuals who fails the first-line treatment but do not seek retreatment will remain in their current state where they may naturally recover or seek treatment in future time steps."

b) "Notation must be changed to improve consistency and clarity. e.g. S is used for number of susceptibles and for S_0 (total number of cases successfully treated); L_0 is used for lifespan but L_1, L_2, for likelihood components (in S3.1). N is used for number treated successfully and for total population size (in S1.2). It is also used for number of simulations in S3.5. Paragraph S3.3, notation problem again, beta used for proportion of cases that are symptomatic (and for transmission rate)."

<u>Response</u>: Thank for point to these double uses of notation. We now use:

- V_0 instead of S_0 to represent the total number of gonorrhea cases successfully treated with Drugs A or B under the status quo;
- E_0 instead of L_0 to represent the effective lifespan of Drugs A and B under the status quo;
- $Z_A(t)$, $Z_B(t)$ and $Z_M(t)$ instead of $N_A(t)$, $N_B(t)$ and $N_M(t)$ to represent the number of gonorrhea cases treated successfully with Drugs A, B, or M during the simulation year t.
- φ_t instead of β_t to represent the proportion of gonorrhea cases in year t of the simulation that are symptomatic.

c) "Results, please indicate what parameters are varied across simulations and inferred (at least the most important ones)"

Response: As described in §S3, all model parameters (except for the population size) are varied across simulations and the parameters listed in Table S2 and Table S4 are inferred through the calibration procedure. We added the following text to Results section to provide more information about the input parameters:

> "Our calibration approach reduced the uncertainty in the estimates for the following parameters (and the correlation between them): transmission parameter, probability that an infection will be symptomatic, duration of infections (without treatment), and time until screened for infection (Table S2 and Fig. S7). Based on the correlation between parameter values and the estimated lifespan of Drugs A and B, the following parameters had an important effects: transmission parameter, duration of infection (without treatment), time until screened for infection, probability that an infection will be symptomatic, and parameters related to the fitness cost of Drugs A and B (Table S9-Table S10)."

(2) "The benefits of spatial strategy explored here of course depends on the spatial heterogeneity in frequency of resistance. This is acknowledged in Discussion. The spatial heterogeneity in resistance is mentioned in "Results". I think this could be emphasised and explained further. The authors could add 1-2 sentences to explain what processes in their model generates heterogeneity in the frequency of resistance (in the Results). For example, variability in the initial frequency of resistance could be a driver of spatial heterogeneity in frequency, is this effect important here? The authors could also plot (as an extra-panel of figure 3 for example) a measure of spatial variation in the frequency of resistance (e.g. variance in frequency as a function of time) in the data, and in the simulations. This would allow a more precise comparison of the extent of spatial heterogeneity in the data and in the simulations."

Response: Thank you for these suggestions.

1. We added the following text to the Result section to clarify the sources of spatial heterogeneity in resistance:

   > "The spatial heterogeneity in resistance in our model is driven by the difference in the rates of gonorrhea cases (Fig. S3), the prevalence of gonorrhea (Fig. S4), and the initial prevalence of resistance to Drugs A and B (Table S5) across different metropolitan areas. This leads to different transmission dynamics in different metropolitan areas and hence, the resistance spreads at a different speed in different areas."

2. We added a new figure (as an extra panel of Fig. 3 and included below) to compare the variance of the prevalence of resistance to Drug A over time among the metropolitan areas of model with the variance of the prevalence of resistance to ciprofloxacin observed in GISP between years 2000 and 2017.

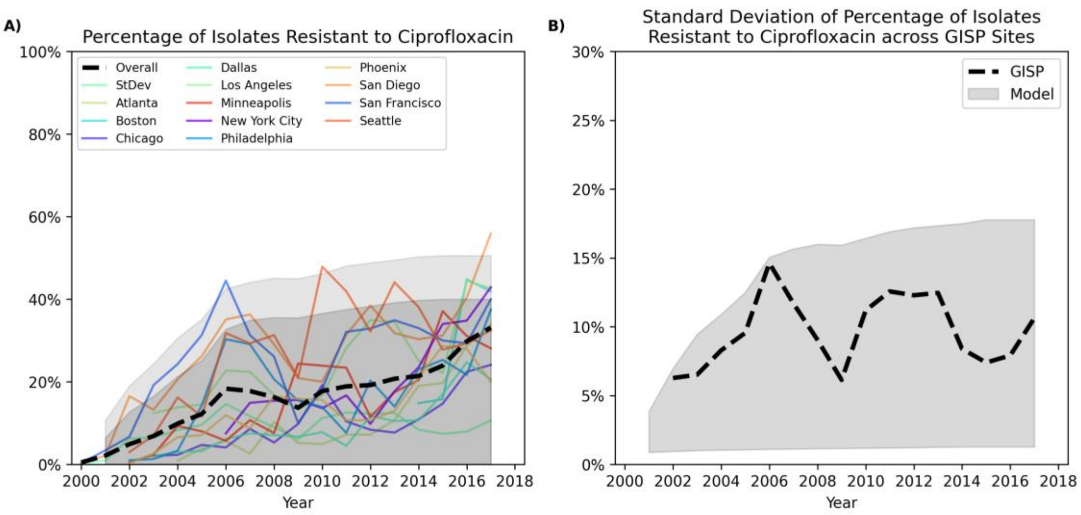

Fig. 3: **Percentage of *Neisseria* gonorrhoeae isolates that are ciprofloxacin-resistant overall and by GISP sites between 2000-2017** [12]. The shaded areas in Panel A represent the 95th (dark grey) and 99th (light grey) percentile interval of the prevalence of resistance to Drug A in the 16 metropolitan areas of our model. Panel B displays the standard deviation for the percentage of isolates resistant to ciprofloxacin across GISP sites. The shaded area represents the 95th percentile interval for the standard division of the prevalence of resistance to Drug A in the 16 metropolitan areas of our model. This figure demonstrates that the rate at which resistance to Drug A increases within the 16 metropolitan areas of our model is consistent with the geographical variation in the trends of ciprofloxacin-resistant gonorrhea observed in GISP.

(3) “Minor points:”

a) “Page 5 need to specify "will seek retreatment with some delay" -> retreatment with the alternative drug, correct? Please specify.”

Response: That is correct. We added a sentence to clarify:

> “A fraction of symptomatic individuals failing the first-line treatment (due to receiving ineffective treatment or developing resistance) will seek retreatment with some delay. These individuals will receive a second-line antibiotic, which is Drug B in the absence of resistance to Drug B and Drug M in the case of resistance to Drug B (Fig. S1).”

b) Page 5 need to give a bit more details on the shape of the declining cost of resistance, and the timescale at which it declines

Response: Thank you for this suggestion. We added a new figure in the Supplement (Fig. S2) to visualize the behavior of function $\gamma_i(t)$, which represents the relative infectiousness of the resistance profile i with respect to the susceptible infection (we note that the fitness cost of resistance declines as $\gamma_i(t)$ increases over time).

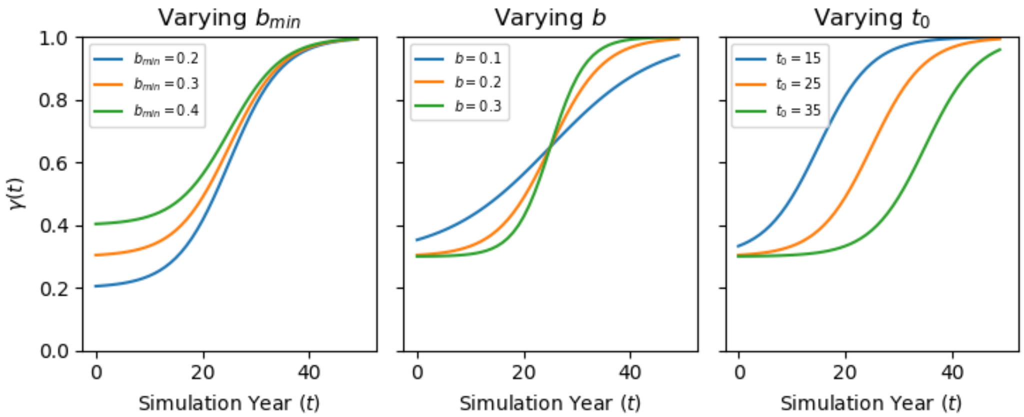

Fig. S1: Behavior of function $\gamma(t)$ (defined in Eq. (2) over time. In these figures, the non-varying parameters are set at the default values $(b_{min}, b, t_0) = (0.3, 0.2, 25)$.

c) Page 6 might be helpful to plot N_A(t), N_B(t) and N_M(t) to give a better intuition of the effective lifespan measure.

<u>Response</u>: Thank you for this suggestion. We have added the plot of these measures in Fig S5 and below:

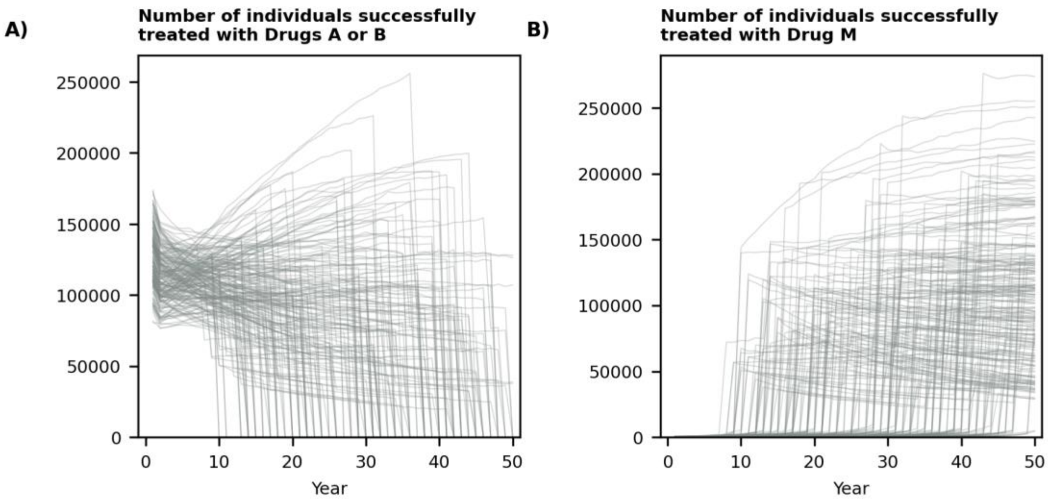

Fig. S2: Number of gonorrhea cases treated successfully with Drugs A or B, and Drug M during the simulation year.

d) Page 6 I was not sure why compute the expected number of additional cases of gonorrhea successfully treated as S_0 * ΔL / L_0. Why not compute it directly from the difference in the results of simulations of the "Base" vs. other strategies? (or, is it actually the same? please specify)

<u>Response</u>: Thank you for raising this question. The Reviewer is correct that for a specified switch threshold, the increase in the effective lifespan of a strategy can be directly measure from the results of simulation. However, as displayed in Fig. 4, an increase in the effective lifespan of a strategy could be

followed by an increase or decrease in the incidence of gonorrhea. Therefore, we thought it would also be helpful to report the maximum increase in the effective lifespan of antibiotics that could be achieved compared to the status quo while keeping the gonorrhea incidence unchanged. This is what ΔE (previously ΔL) is estimating, which is essentially the horizontal distance between the points where the curves in Fig. 4A crosses the x-axis. After estimating ΔL, we then use the formula $V_0 \frac{\Delta E}{E_0}$ to also estimate the expected number of additional cases of gonorrhea that could be treated without changing the number of gonorrhea cases. We made the following changing to clarify:

> "<u>As we will demonstrate later, changing the switch threshold from 5% would impact both the effective lifespan of Drugs A and B and the number of annual gonorrhea cases. Therefore, we define ΔE as the maximum increase in the effective lifespan of antibiotics that could be achieved compared to the status quo while keeping the gonorrhea incidence unchanged.</u> For any strategy that extended the effective lifespan of Drugs A and B by ΔE years, we estimated the expected number of additional cases of gonorrhea that would be treated successfully with first-line antibiotics <u>without increasing the gonorrhea cases</u> during the simulation period with $V_0 \frac{\Delta E}{E_0}$, where V_0 is the total number of cases successfully treated with Drugs A or B during the simulation period, and E_0 is the effective lifespan of Drugs A and B under the status quo [15]."

e) Page 7 please define the intervals given in parentheses e.g. "200 (169, 232)"

<u>Response</u>: The intervals represent the 95% uncertainty intervals, as described under the subsection "Comparing the performance of treatment guideline strategies". We also made this explicit the first time we report an interval in the Result section.

> "We summarized results using the mean and 95% uncertainty interval (i.e., the interval between 2.5th and 97.5th percentiles of realizations) across 500 simulated trajectories."

f) Page 10 "Incorporating the exact prevalence of gonorrhea in each city could improve the predictive power of the model but is not expected to influence the comparative performance of strategies simulated here" -> I believe variability in prevalence across cities (particularly if correlated with frequency of resistance) could change the quantitative impact of the different strategies (figure 4). This seems important to note, as the quantitative effects are an important part of the results.

<u>Response</u>: We apologize for causing confusion. In response to a reviewer's comment on our initial submission, we partially relaxed this assumption but didn't update the Discussion section. While estimates for the prevalence of gonorrhea are not available for all 16 metropolitan areas, we found such estimates for five metropolitan areas (Houston, Miami, New York, San Francisco, and Washington, DC). The model is calibrated to the prevalence estimates from these areas (Fig S3). For other areas where such estimates are not available, for each simulated trajectory, we determined the initial prevalence of gonorrhea by random draws from appropriate probability distributions (Table S4). Therefore, while incorporating the exact prevalence of gonorrhea in each city could improve the predictive power of the model, we believe that our conclusions are robust to this source of uncertainty. We updated the discussion section accordingly:

"Second, data on gonorrhea prevalence were only available for five metropolitan areas (Houston, Miami, New York, San Francisco, and Washington, DC) (Fig. S4). For each simulated trajectory, we estimated the initial prevalence of gonorrhea in areas without locally available prevalence data by random draws from appropriate probability distributions (Table S4). While this allowed us to confirm that our conclusions were robust over a range of plausible prevalence levels, limited local prevalence data precludes precise prediction of the magnitude of benefits of spatially-adaptive policies."

g) Not clear about the role of diagnosis of symptomatic / asymptomatic cases in relation to the model as explained in S1.2. Is "diagnosed" equivalent to "waiting to receive first line treatment"?

Response: That is correct. We have assumed that cases who are symptomatic will seek treatment and those who are asymptomatic can be detected through screening and move to the W's and W''s compartments while waiting to receive treatment or retreatment. We have specified this in the definitions of compartments $W_{(k,i,s)}(t)$ and $W'_{(k,i,s)}(t)$ in §S1.2:

- "$W_{(k,i,s)}(t)$: number of diagnosed cases with the resistance profile i, waiting to receive the first-line therapy in the metropolitan area k at time t;
- $W'_{(k,i,s)}(t)$: number of diagnosed cases with the resistance profile i, waiting to receive the second-line therapy in the metropolitan area k at time t."

h) S1.2 typo in the list of possible k ("m16" instead of ",16")

Response: Thank you for identifying this error.

i) Why isn't the estimated fraction of infections that are symptomatic (49.2%, table S2) close to the fraction of symptomatic in the data (67.9%)?

Response: Table S2 reports the "probability that a new infection will be symptomatic," which is an input parameter of our simulation model to determine what portion of new infections will be symptomatic. The estimated reported by the data (as used in Fig. 2C) is the fraction of detected cases that symptomatic. We expect these two estimates to be difference since many asymptomatic infections will never get detected.

j) Typo at the end of S3.4 "because that" (remove "that")

Response: Thank you. We fixed the typo.

k) S3.5 I don't get the point of removing L_max in the expression from the weight, doesn't exp(-L_{max}) cancel out? If the authors agree, then there just remains weight proportional to L_i / sum(L_i).

Response: The Reviewer is correct that mathematically, $e^{L_{max}}$ gets cancelled out. This term is added for numerical reasons. We have added the following sentences to clarify:

"We note that since $\ln \mathcal{L}_i$ could be a negative number, $e^{\ln \mathcal{L}_i}$ might be calculated as 0 in computer even though mathematically, $e^{\ln \mathcal{L}_i} > 0$ for any $\ln \mathcal{L}_i > -\infty$. A common approach to mitigate this

issue is to subtract L_{max} from all likelihoods $\ln \mathcal{L}_i$. This doesn't change the estimated w_i's but it makes the estimates more stable numerically."

l) I do not understand the difference between prior / posterior distributions of parameters, versus "uncertainty range" / "feasible interval" used for parameters related to emergence of resistance (table S3). The criterion of reaching at least 5% resistance can also be formulated as a pseudo-likelihood function with a particular form (i.e. probability 1 if 5% resistance is reached for both drugs, 0 otherwise).

Response: Thank you for raising this question. We agree with the Reviewer that the criterion of reaching at least 5% resistance can easily be incorporated into the pseudo-likelihood function. The reason that we chose to use "uncertainty range + feasible interval" as opposed to "prior + posterior distributions" was to address the concern raised by other reviewers. Since Drugs A and B are hypothetical future antibiotics, some believe that "prior distributions" cannot be specified for parameters related to these drugs. We have discussed this in §3.5 but agree with the Reviewer that mathematically, these two approaches are equivalent:

"As Drug A and Drug B are hypothetical future antibiotic treatments for gonorrhea, we did not specify prior distributions for the parameters that relate to the emergence and spread of resistance to these drugs. Instead, we derived relevant ranges for these parameters (Table S3 and Table S5-Table S6) by retaining only those simulated trajectories where the prevalence of resistance to Drug A and Drug B reaches at least 5% during simulations."
